# Metabolomic Mapping of Greek Olive Leaves by Untargeted NMR‐Based Profiling and LC–HRMS Dereplication

**DOI:** 10.1002/ansa.70094

**Published:** 2026-06-30

**Authors:** Mariacaterina Lianza, Stavros Beteinakis, Vasileios Siderakis, Panagiotis Stathopoulos, Emmanuel Hatzakis, Maria Halabalaki

**Affiliations:** ^1^ Division of Pharmacognosy and Natural Products Chemistry Department of Pharmacy National and Kapodistrian University of Athens Athens Greece; ^2^ Department of Food Science and Technology The Ohio State University Columbus Ohio USA

**Keywords:** ^1^H NMR profiling, chemotaxonomic markers, LC–HRMS, *Olea europaea*, Oleaceae, oleuropein, olive leaves, pentacyclic triterpenoids

## Abstract

Olive tree leaves are phytochemically rich yet insufficiently valorised in terms of systematic cultivar‐based characterisation and exploitation, and are an abundant olive industry side‐product. This study represents the first large‐scale metabolome mapping based on 340 leaf extracts from five major Greek cultivars (Amfissis, Koroneiki, Manaki, Lianolia‐Kerkyra and Thasou) collected across diverse regions, agronomic practices, harvest periods and years. An integrated analytical workflow combining untargeted ^1^H NMR profiling and LC–HRMS dereplication was applied for the first time at this scale. NMR provided a quantitative and highly reproducible overview of the core metabolome and served as the basis for multivariate statistical analysis, while HRMS enabled confident structural annotation of 62 metabolites, including secoiridoids, flavonoids, phenolic acids, triterpenoids and fatty acids. Multivariate data analysis (PCA and OPLS‐DA) revealed that cultivar identity constitutes the dominant driver of metabolic variation, exceeding the influence of environmental, agronomic and temporal factors. Pentacyclic triterpenic acids, that are, oleanolic and maslinic acids, emerged as robust, genotype‐dependent chemotaxonomic markers, outperforming the widely used oleuropein, whose abundance is highly sensitive to environmental and agronomic factors. Flavonoids (apigenin, luteolin, quercetin) and the sugar alcohol mannitol displayed cultivar‐specific patterns consistent with differential stress adaptation strategies rather than strict taxonomic control. Amfissis was characterised by the highest and most stable triterpenoid levels, Koroneiki/Thasou by elevated flavonoid levels, while Koroneiki/Manaki showed the highest relative concentration of oleuropein and mannitol. Overall, this integrated NMR/LC–HRMS approach establishes a robust framework for olive leaf cultivar authentication and supports genotype‐driven valorisation of this agricultural side‐product, enabling the development of standardised, cultivar‐specific extracts for phytotherapeutic, nutraceutical and cosmetic applications.

## 1 Introduction

Olive tree leaves have long been employed in Mediterranean traditional medicine for their therapeutic value. Historical records indicate that preparations from olive leaves were already used in ancient Egyptian and Greek medicine for the treatment of fever, infections and inflammatory conditions [1, 2]. Modern phytochemical investigations have confirmed that these biological effects are associated with the presence of several bioactive compounds, which are responsible for the antinociceptive, anticancer [3], cardioprotective [4] and neuroprotective properties [5] exhibited by olive leaf extracts. Based on the current knowledge, olive leaves contain secoiridoids, particularly oleuropein and its derivatives, as well as simple phenols, such as hydroxytyrosol and tyrosol [6]. These bioactive compounds have demonstrated a range of biological activities, including antimicrobial [7], anticancer [8], anti‐inflammatory [9] and anti‐atherogenic [10] actions, together with hypolipidemic and hypoglycaemic effects [11]. Several phenylethanoid glycosides found in the leaves, such as verbascoside, are also accountable for marked anti‐inflammatory activity, in addition to antioxidant, wound‐healing and immunomodulatory properties [12, 13]. Triterpenoids, such as oleanolic and maslinic acids, are abundantly present in the outer coating of leaves [14]. These metabolites are endowed with hepatoprotective [15, 16] and antitumour [14] activities. Olive leaves also contain flavonoids, including apigenin, luteolin and their glycosidic forms, which contribute to the radical scavenging capacity and modulation of inflammatory pathways [17, 18]. Different types of sugars and organic acids have also been reported [19].

Despite their phytochemical richness, olive leaves are generated in large quantities as an olive industry side‐product and are not yet valorised proportionally to their availability, especially compared with the extensively exploited olive fruit. Olive oil production generates substantial quantities of by‐products or side products, including leaves, accounting for approximately 10% of the total olive harvest mass [20]. Furthermore, the considerable biomass of leaves generated during olive tree pruning, typically in December and March, is often disposed of by burning, fermenting or composting, which can lead to economic and environmental issues. Therefore, olive leaf valorisation is crucial for advancing the principles of the circular economy within the olive industry.

The presence of the aforementioned bioactive compounds has led to a growing interest in repurposing this by‐product into high‐value ingredients for various industries, namely, food, cosmetics and nutraceuticals, representing a promising avenue for sustainable resource utilisation. Moreover, several olive leaf applications have been developed, such as their use as additives in food processing and preservation [21] and as antioxidant and antimicrobial agents [20]. In addition, olive leaf metabolite profiles can indicate plant health or disease and can be used to track metabolic responses following agronomic or other treatments [19]. Therefore, knowledge about their composition could also be beneficial for plant protection purposes. Although several studies have been conducted on the composition of olive leaf extracts, most have focused on a limited number of samples and targeted selected compounds for specific functions or purposes. Comprehensive mapping of the olive leaf metabolome remains necessary to fully elucidate the biochemical diversity among cultivars and to identify robust chemical markers for varietal traceability, agronomic monitoring and extract standardisation. Multi‐technique metabolomic strategies combining ^1^H nuclear magnetic resonance (NMR) with complementary mass spectrometric platforms have recently been shown to improve authentication workflows in plant matrices [22, 23], and no study to date has systematically integrated large‐scale untargeted ^1^H NMR metabolomics with liquid chromatography coupled to high‐resolution mass spectrometry (LC–HRMS) dereplication to compare Greek olive leaf cultivars under diverse agronomic and environmental conditions. To address this gap, 340 olive leaf samples were collected across Greece, an unprecedented number in such an investigation. These samples belong to the five main Greek cultivars, spanning different geographical areas, subjected to different farming systems, and collected in different time periods. The aim of this study is twofold: first, to achieve an in‐depth chemical characterisation of the major Greek olive leaf cultivars and, second, to determine whether and how cultivar identity constitutes a dominant source of metabolic variation. To this end, untargeted ^1^H NMR metabolomics was integrated with LC–HRMS dereplication to provide a comprehensive and structurally informed metabolic assessment. By combining compositional profiling with both unsupervised and supervised chemometric analyses, this work defines cultivar‐associated metabolic signatures and evaluates their discriminatory capacity within a chemotaxonomic framework. Ultimately, this integrated approach establishes a robust basis for linking metabolic phenotypes to botanical origin and supports future applications in authentication, breeding strategies and optimised valorisation of olive leaf biomass.

## 2 Experimental

### 2.1 Sample Collection

**FIGURE 1:**
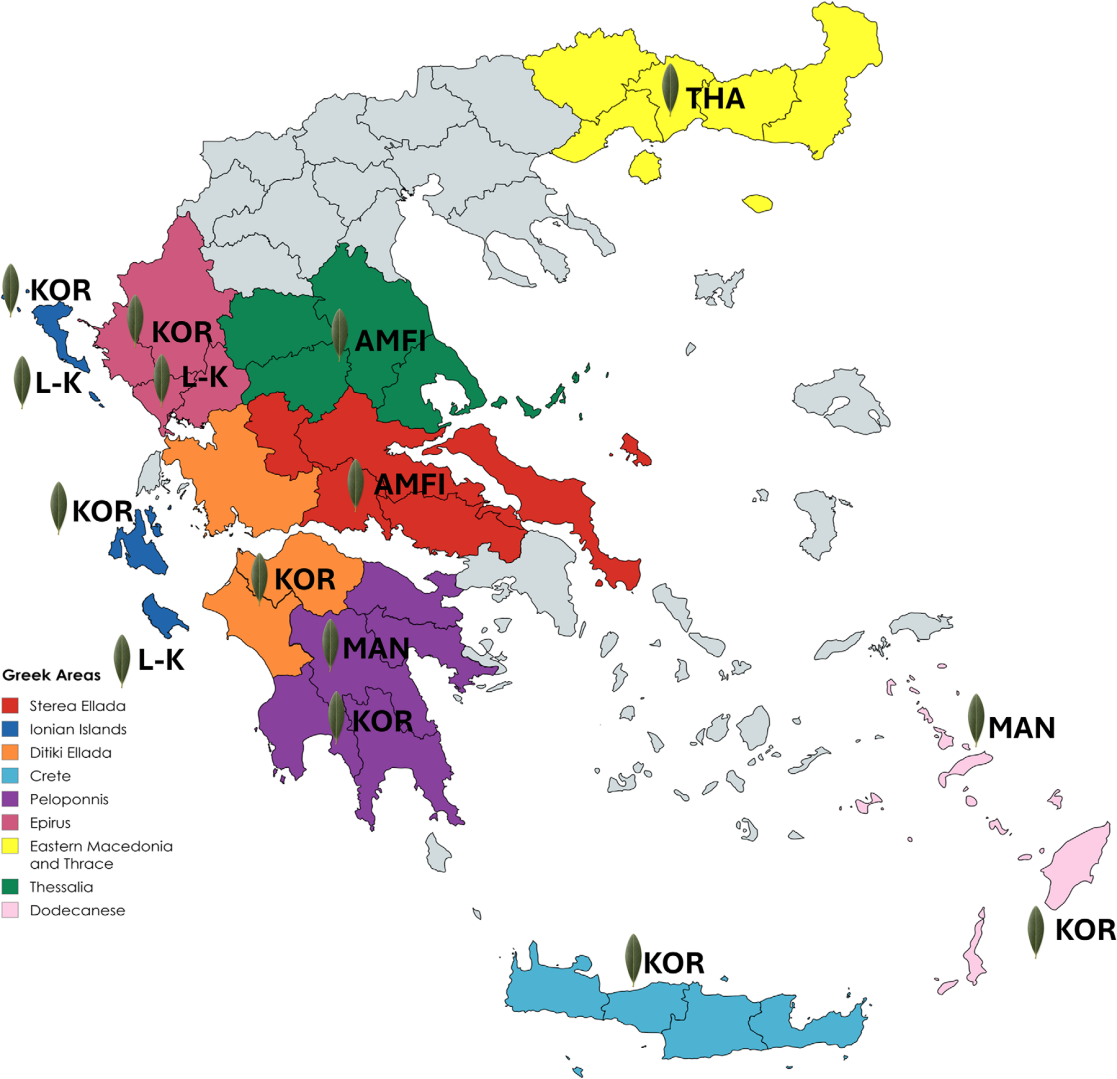
Sample collection map across Greece. Olive leaf samples under different cultivation conditions belonging to five cultivars, namely, Amfissis (AMFI), Koroneiki (KOR), Lianolia‐Kerkyra (L‐K), Manaki (MAN) and Thasou (THA), were collected in different areas of the country over 3 years.

Three hundred forty olive leaf samples from five cultivars, namely, Amfissis (AMFI), Koroneiki (KOR), Manaki (MAN), Lianolia‐Kerkyra (L‐K) and Thasou (THA), were collected across different geographical areas in Greece (Crete, Dodekanisa, Dytiki Ellada, Eastern Macedonia, Epirus, Ionian Island, Peloponnese, Sterea Ellada, Thessalia, see Figure 1). The leaves were derived from olive trees cultivated either with conventional or organic farming methods, under regular irrigation or not. The collection period covered 3 years, from 2019 to 2021, with a sampling date located in two periods of the year (January–September or February–August). Detailed metadata are listed in Table S1.

### 2.2 Chemicals

The methanol used for extraction was high‐performance liquid chromatography (HPLC) grade and was purchased from Fisher Scientific (Leicestershire, UK). Deuterated methanol (methanol‐*d*_4_, 99.8% D) for NMR analysis was acquired from Merck SA (Athens, Greece), while the internal standard (IS) hexamethyldisiloxane (HMDSO, NMR grade) was acquired from Sigma‐Aldrich Corporation (St. Louis, MO, USA). Acetonitrile (ACN) of liquid chromatography‒mass spectrometry (LC‒MS) grade (LiChrosolv, Hypergrade) was obtained from Merck SA, while LC‒MS grade MeOH and formic acid were supplied by Fisher Chemical (Loughborough, UK). Ultrapure water for LC‒MS analysis (18.2 MΩ cm) was produced by a Milli‐Q purification system (Merck Millipore, Darmstadt, Germany).

### 2.3 Sample Extraction

Sample preparation followed the procedure described in the European Pharmacopoeia [24]. One gram of naturally dried and powdered olive leaves was extracted with 50 mL of MeOH for 30 min at 60°C. The extract obtained was diluted to a final volume of 100 mL, and an aliquot of 5 mL was recovered for NMR analysis. After filtration, the solvent was evaporated under reduced pressure to obtain the dried olive leaf extract. As extract yields were affected by sample variability, the exact mass of each aliquot also varied. For calculation purposes, each aliquot was considered equivalent to one twentieth of the total extract, corresponding to approximately 50 mg of dried olive leaves.

### 2.4 NMR Analysis

#### 2.4.1 Sample Preparation

A stock solution of methanol‐*d*_4_ with HMDSO as the IS and a line‐shape indicator (0.02% v/v) was prepared. Each sample was dissolved in 700 µL of the deuterated stock solution, filtered through a 0.45 µm PVDF filter and transferred into a 5 mm NMR tube (D600, Deutero GmbH, Kastellaun, Germany) with a PTFE cap (Deutero GmbH).

#### 2.4.2 NMR Acquisition Parameters

1D NOESY NMR experiments were recorded at 305 K on a Bruker AVANCE III 600 NMR spectrometer (Bruker GmbH, Rheinstetten, Germany) equipped with a 5 mm inverse probe with *z* gradients and a Bruker Control Unit (BCU) for temperature control. A 60‐place sample charger (B‐ACS‐60) controlled with the IconNMR software by Bruker was used for automation. ^1^H NMR spectra were obtained with the ‘noesygppr1d’ pulse sequence using an ∼8 µs (π/2) pulse, number of scans 128, spectral width of 12,019.2 Hz, time domain (TD) 64k data points, acquisition time of 2.73 s and relaxation delay of 2 s. The TD data were Fourier transformed by applying exponential multiplication with a line‐broadening factor (lb) of 0.3 Hz and zero‐filling (size = 128K). Phase and baseline corrections were conducted automatically using TopSpin by Bruker (version 4.2.0). Spectra were referenced with the IS signal set at *δ*_H_ 0.00. The 1D NOESY spectra were used for metabolomic profiling. Identification of compounds was accomplished through two‐dimensional (2D) NMR spectra (COSY, HSQC‐DEPT, HMBC, JRES) on selected samples and comparison with in‐house data and literature.^13^C chemical shifts reported in Table S2 were obtained from the aforementioned HSQC‐DEPT spectra.

#### 2.4.3 Computational Processing of NMR Data and Chemometric Analysis

Raw ^1^H NMR spectra were converted into datasheets/numerical data for further processing using the MATLAB suite (version R2024b, MathWorks, Natick, MA, USA). First, alignment of the raw data was accomplished using the icoshift tool (Version 3.0) and selected intervals [25]. Then, NMR spectra (spectral width from *δ*_H_ 11.2 to −0.2) were segmented into equal‐sized bins with a bin size of 0.01 ppm. Solvent peaks, IS signals and baseline noise were removed. Multivariate analysis (MVA) was accomplished within the SIMCA environment (v. 18.0.0.372, Sartorius AG, Göttingen, Germany). A principal component analysis‐class (PCA‐class model) was carried out using 340 observations and 1074 X‐variables, Pareto scaled and subjected to log transformation to reduce skewness and stabilise the variance, improving normality and homoscedasticity. Analysis of variance (ANOVA) was performed to assess the significant differences between the sample groups. Tukey's HSD post hoc tests were used for multiple comparisons of means (*p* < 0.05). The same work set was employed to carry out an orthogonal partial least squares discriminant analysis (OPLS‐DA model) considering the botanical origin as the *Y* variable. To formally assess whether the OPLS‐DA model significantly explained the variance in the data, a cross‐validation ANOVA (CV‐ANOVA) was performed. The *F* values were calculated as the ratio between the regression mean square and the cross‐validated residual mean square, providing a statistical test of class discrimination. The model was also rigorously validated by performing a permutation test with 500 permutations for each binary comparison (Table S4). Violin plots were employed to visualise the ANOVA data. Univariate analyses were carried out using GraphPad Prism 9.0.0 (GraphPad Software Inc., CA, USA).

### 2.5 LC–HRMS/MS Analysis

Samples were dissolved in MeOH at a concentration of 300 µg/mL. Chromatographic analysis was performed on a Vanquish Flex Ultra‐High‐Performance Liquid Chromatography (UHPLC) system coupled with an Orbitrap Exploris 120 mass spectrometer (Thermo Scientific Inc., USA) with a heated electrospray ionisation (HESI‐ІІ) source operating in both positive and negative ion mode. Xcalibur software v. 4.7.102.25 (Thermo Scientific) was used for data acquisition and processing. Chromatographic separation was conducted on an Acquity BEH C18 column (150 × 2.1 mm, 1.7 µm) (Waters Corporation, Milford, MA, USA). The column temperature was set at 40°C, while the autosampler compartment was thermostated at 7°C. A gradient elution program was employed comprising water with 0.1% (v/v) formic acid (Solvent A) and ACN (Solvent B), delivered at a flow rate of 0.3 mL/min. Elution began with 95% A for the first 2 min, and a gradient from 95% to 85% A was set in the next 3 min. In the following 15 min, the composition of A was further decreased to 60% and to 0% in another 8 min. It was then held isocratically for 1 min. Finally, the initial mobile phase composition was restored over the next 1 min and maintained for the last 2 min for column re‐equilibration. The total runtime was 30 min. The injection volume was 5 µL.

Mass spectra were obtained using an *m*/*z* range of 113–1300 Da for full scan acquisition at a resolving power of 60,000 (full width at half maximum, FWHM, at *m*/*z* 200). High‐resolution tandem mass spectrometry (HRMS/MS) spectra were recorded using data‐dependent acquisition (ddMS2) with normalised HCD collision energies of 30%, 50% and 150% and a resolving power of 30,000 at 200 *m*/*z*. The optimal HRMS parameters chosen were the following: capillary and source heater temperatures were set at 350°C, source voltage at 3.5 kV for negative and 3.8 kV for positive ion modes, respectively, and S‐lens RF level at 60%. Sheath and auxiliary gases were appointed at 45 and 20 arbitrary units, respectively.

Accurate‐mass full‐scan data acquired in both negative and positive ionisation modes were used to propose candidate molecular formulas, supported by isotope pattern fit and ring double bond equivalent (RDBeq) values. Targeted interrogation of data‐dependent MS/MS spectra enabled structural annotation by combining precursor *m*/*z*, adduct type, diagnostic fragment ions and characteristic neutral losses. Spectral interpretation and tentative identification of metabolites were supported by (i) an in‐house library of previously characterised *Olea* metabolites, (ii) curated spectral databases (mzCloud, NIST, MassBank) and (iii) literature reports for olive leaves and related matrices [26, 27, 28, 29, 30, 31, 32]. Retention behaviour and ionisation/adduct patterns were additionally considered to support compound class assignment and to assist the interpretation of isomeric signals.

## 3 Results and Discussion

### 3.1 Compositional Analysis of Olive Leaf Methanol Extracts

**FIGURE 2:**
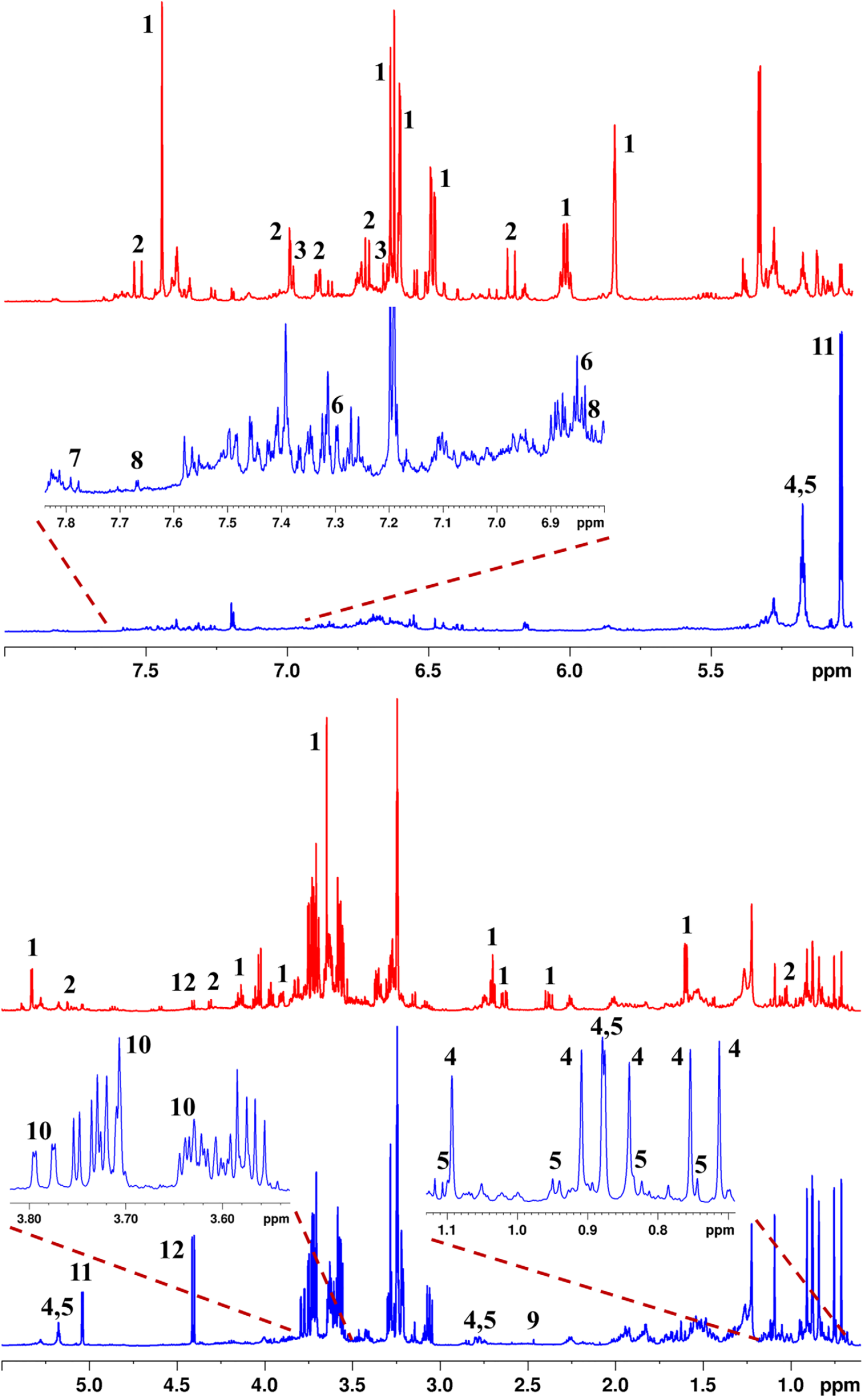
Representative 600 MHz ^1^H NMR spectra of two methanol olive tree leaf extracts in different spectral regions, from *δ*_H_ 0.6 to 8.0, with the major metabolites annotated. (1) Oleuropein; (2) verbascoside; (3) ligstroside; (4) oleanolic acid; (5) maslinic acid; (6) luteolin; (7) apigenin; (8) quercetin; (9) succinic acid; (10) d‐mannitol; 11: α‐glucose; (12) β‐glucose.

NMR spectroscopy provides highly reproducible spectral fingerprints, linear and inherently quantitative responses and minimal instrumental variability across runs and batches. Therefore, NMR data are particularly suitable for MVA aimed at exploring metabolomics trends and cultivar‐dependent differences [33]. The ^1^H NMR spectra of the olive leaf methanol extracts were characterised by numerous signals belonging to both primary and secondary metabolites. The aromatic zone (*δ*_H_ 6.0–8.0) was dominated by resonances attributable to phenolic compounds and secoiridoids. In most of the olive leaf samples, oleuropein turned out to be the dominant compound, in agreement with previous studies [6]. However, in several samples, the oleuropein resonances were markedly reduced, consistent with literature reports indicating a wide variability of this secoirodoid in olive leaves, with reported levels ranging from approximately 1%–14% of the dry weight, depending on cultivar, harvest period, environmental conditions and extraction technique [34]. In these samples, signals attributable to the oleosidic protons of ligstroside became discernible. Oleuropein and ligstroside display highly similar ^1^H NMR spectral features, particularly within the sugar and secoiridoid regions, leading to extensive signal overlap in crude extracts. In olive leaf matrices, where oleuropein typically represents the dominant secoiridoid, the identification of ligstroside may therefore be hampered by signal superposition, rendering its unambiguous assignment challenging without complementary MS‐based evidence [35]. In the mid‐field region (*δ*_H_ 3.0–5.5), the spectra displayed intense signals corresponding to sugars and glycosidic moieties. The aliphatic region (*δ*_H_ 0.6–2.5) was characterised by intense singlets and multiplets attributable to pentacyclic triterpenoids, which represented the predominant constituents in many of the analysed extracts. Figure 2 shows the major compounds of the olive leaf extracts as identified by using an in‐house library of isolated olive compounds, 2D NMR analysis and literature. In addition to the secoridoids oleuropein and ligstroside and the phenylethanoid glucoside verbascoside, several other compounds were identified. These include two triterpenoids, namely, oleanolic and maslinic acids, the two flavones luteolin and apigenin, the flavonol quercetin, succinic acid, the free carbohydrates β‐glucose and its α‐anomer, and the sugar alcohol mannitol. Table S2 lists the diagnostic and representative signals supporting these assignments. The assignments should be interpreted as metabolomic annotations in complex extracts rather than full structural elucidation of each compound. Despite the differences in concentration due to many factors, such as variety, climate conditions, tree age and many others, several investigations have identified the listed compounds among the most typical for olive leaves [1, 6, 36].

### 3.2 Multivariate Data Analysis

To obtain an overview of the data, an NMR‐based PCA was carried out as an exploratory tool with the aim of identifying any patterns in the dataset, reducing the data complexity and visualising differences among the samples. In the PCA, formed by 28 principal components, the botanical origin was set as class, while the geographical areas of harvesting, the tree age, the farming method, the type of irrigation, the collection year and the collection period were set as secondary observations.

**FIGURE 3:**
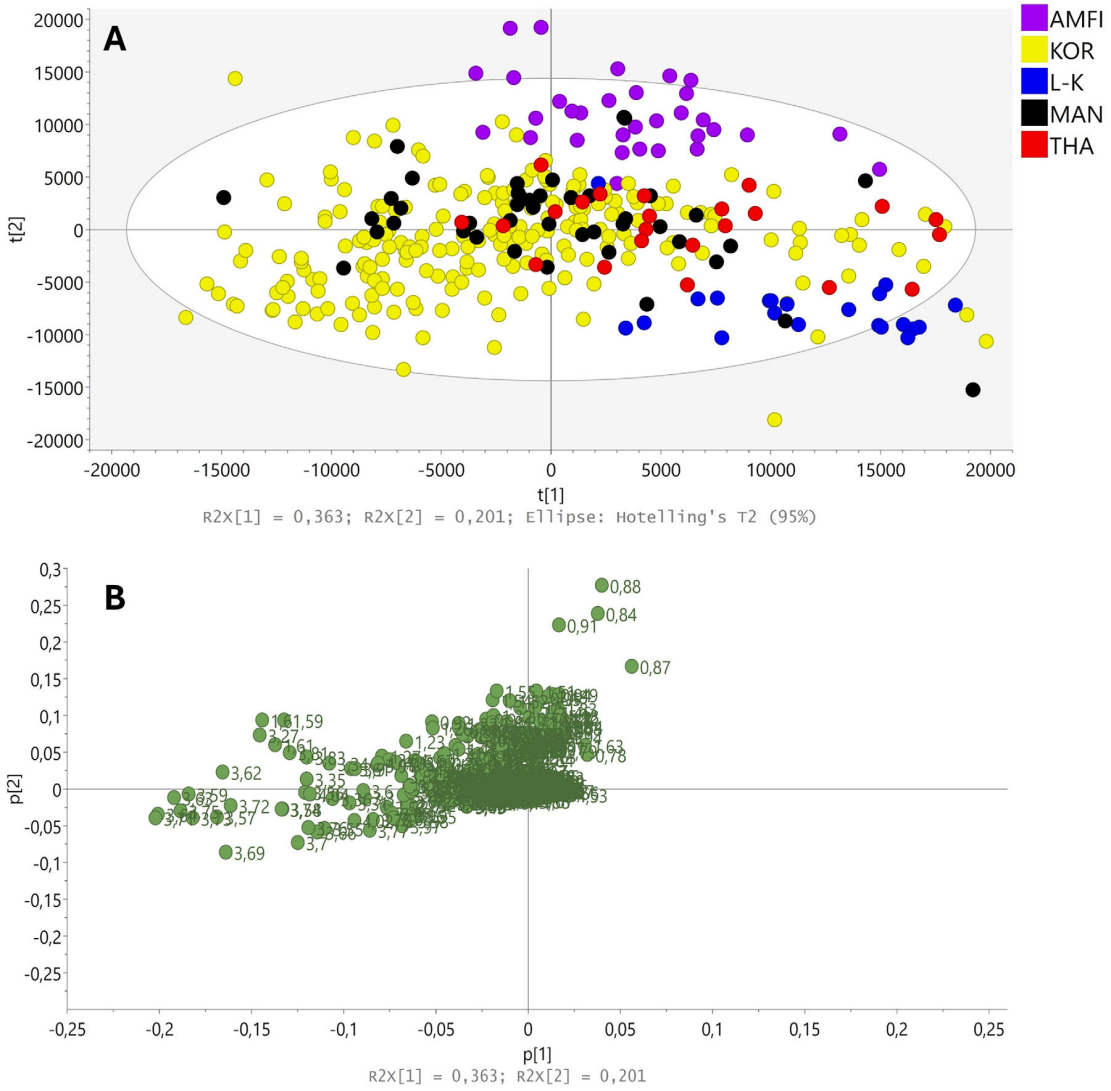
(A) PCA score scatter plot. PC1 explained 36.3% of the variation, while PC2 explained 20.1%. The 340 olive leaf extract samples were coloured by cultivar type: AMFI, KOR, L‐K, MAN and THA. Neighbouring samples showed similar chemical composition; a partial clustering is visible according to the cultivar (R^2^X (cum) = 0.97 and Q^2^ (cum) = 0.93). (B) PCA loading plot. The bins *δ*_H_ 0.84, 0.87, 0.88 and 0.91 mainly drove the sample distribution along t[2], while the ^1^H NMR signals at *δ*_H_ 1.59‐1.61 and *δ*_H_ 3.59‐3.73 mostly influenced the sample distribution along t[1].

The PCA score plot in Figure 3A revealed a partial but discernible cultivar‐dependent clustering of the samples. PC1 explained 36.3% of the total variance, while PC2 accounted for 20.1%, together describing more than half of the overall metabolic variability. AMFI samples were predominantly located in the upper region of the plot, while L‐K samples clustered mainly in the bottom right quadrant. MAN and THA occupied more central positions, showing partially overlapping distributions, although MAN tended to shift toward the left quadrants, while THA was more represented in the right quadrants. KOR exhibited the highest intra‐cultivar dispersion, spanning multiple regions of the score plot, consistent with its broader geographical representation and larger sample size. The corresponding loadings plot (Figure 3B) clarified the variables responsible for this separation. Based on the compositional analysis previously conducted, the bins at *δ*_H_ 0.84, 0.87, 0.88 and 0.91, which strongly drove the distribution in the upper quadrants, were assigned to the methyl resonances of the pentacyclic triterpenoids (oleanolic and maslinic acids). Hence, AMFI was the cultivar with the highest relative concentration of triterpenoids, revealing a distinctive feature. Most of the resonances that contributed to the displacement of samples toward t[1] (i.e., *δ*_H_ 1.59‐1.61 and *δ*_H_ 3.59‐3.73) were attributed to oleuropein and mannitol, respectively. Notably, these metabolites exhibited distinct distribution patterns, with the samples with the highest relative oleuropein signal intensities positioned in the upper left quadrant and the samples with the highest relative mannitol signal intensities in the bottom left quadrant. The PCA model did not highlight any outstanding correlation between the sample chemical profiles and the year of collection, farming method, tree age, irrigation mode, geographical area or collection period (Figure S1). The outliers, namely, the samples outside Hotelling's T^2^ ellipse (95%), marking the boundary for normal multivariate variation, represented either biological extremes or technical variations in sample processing. Since the number of outliers was limited compared to the large number of samples belonging to the dataset, the overall model quality was not compromised.

**FIGURE 4:**
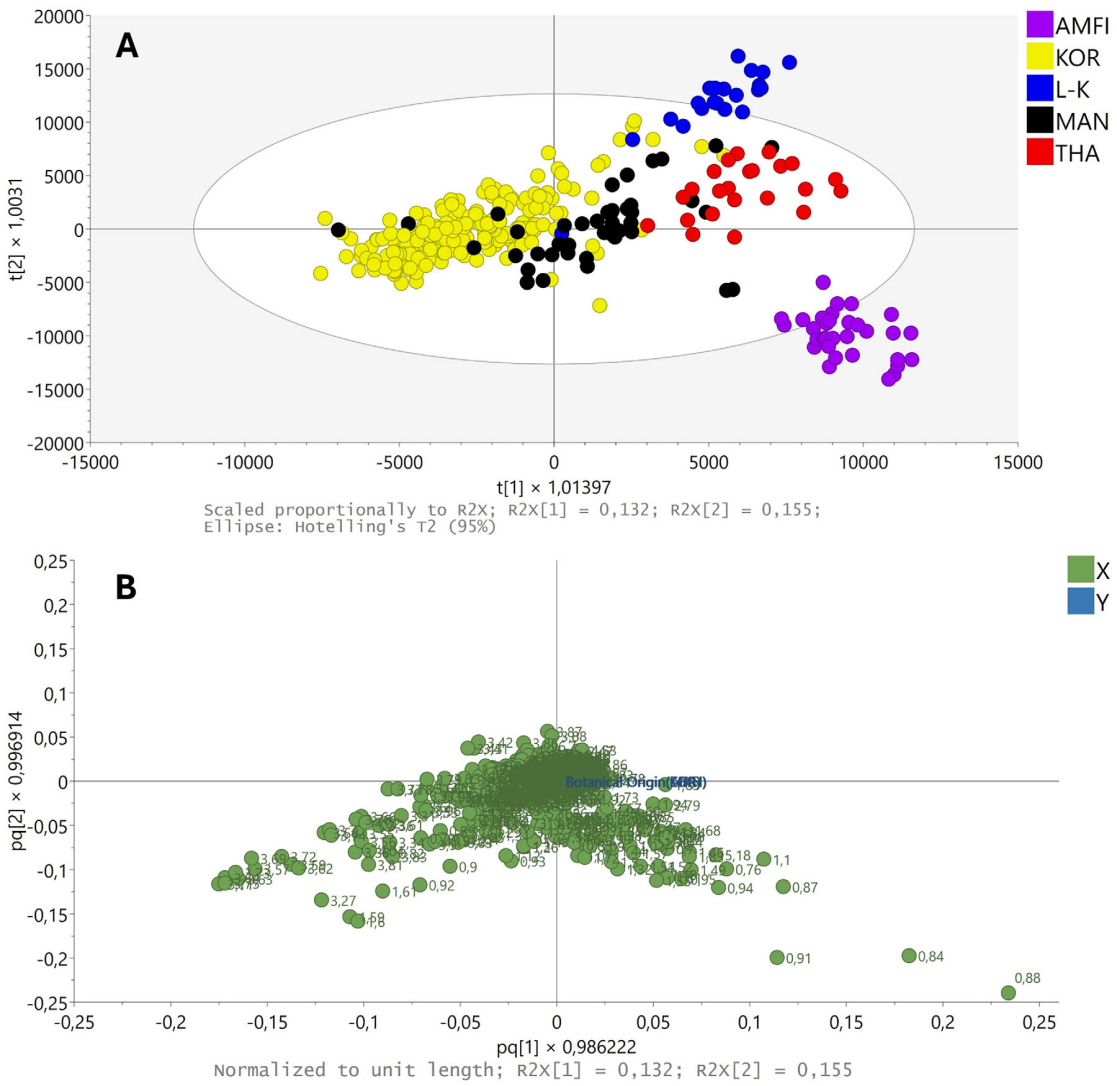
(A) OPLS‐DA score scatter plot obtained from the ^1^H NMR data of the olive leaf extracts using cultivar as the *Y*‐variable. Olive leaf samples are coloured according to their botanical origins: AMFI, KOR, L‐K, MAN and THA. The model showed the clustering of samples according to their botanical origin, indicating cultivar‐dependent metabolomic fingerprints. (B) OPLS‐DA loading plot. Each dot represents an *X* variable, with the associated number indicating the corresponding NMR bucket responsible for class separation. Signals located farthest from the center contribute most to the discrimination among cultivars.

The PCA model pointed out that botanical origin represented the main source of variation among the olive leaf samples; therefore, an OPLS‐DA model was developed considering the cultivar as the *Y* variable (Figure 4). This supervised multivariate approach allows the decomposition of the total variance in the NMR dataset into two distinct parts: a predictive component, directly correlated with the classification factor (botanical origin), and orthogonal components, which account for systematic but unrelated variation. By maximising the covariance between *X* (spectral data) and *Y* (cultivar), OPLS‐DA enhances group separation and facilitates the interpretation of class‐related metabolic differences.

In this model, the variance percentages explained by the *X* variables (*R* ^2^ *X* (cum)) and the *Y* variable (*R* ^2^ *Y* (cum)) were 87% and 70%, respectively, indicating a high capture of information from the metabolomic space and a solid correlation between the metabolomic profile and the botanical origin. The predictivity capacity *Q* ^2^ (cum) was 0.63, denoting a robust model with limited overfitting. The cross‐validation ANOVA (CV‐ANOVA; Table S3) showed *p* < 0.001 and regression mean squares between 8.3 and 14.5, markedly higher compared to the residuals (0.15–0.53), confirming that all the cultivar models are highly statistically significant; thus, the observed separation among the botanical origins is not attributable to random variation. The *F* values ranged between 15.2 and 93.7, while the standard deviation of the cross‐validated residuals (SD), reflecting their dispersion, was between 0.39 and 0.73. AMFI, as previously indicated by the PCA, emerged as the most distinctive and consistent metabolomic fingerprint (*F* = 93.7, SD = 0.39). It was followed by KOR (*F* = 42.5, SD = 0.54), showing great internal consistency and intermediate variability, in line with its wide geographical distribution and sample size. L‐K and THA (*F* = 15.1, SD = 0.73; *F* = 20.4, SD = 0.68, respectively) showed a discrete separation and more heterogeneous metabolomic profiles compared to the abovementioned cultivars, while MAN (*F* = 16.2, SD = 0.72) was confirmed as having an intermediate chemical profile, overlapping the other botanical origins.

**FIGURE 5:**
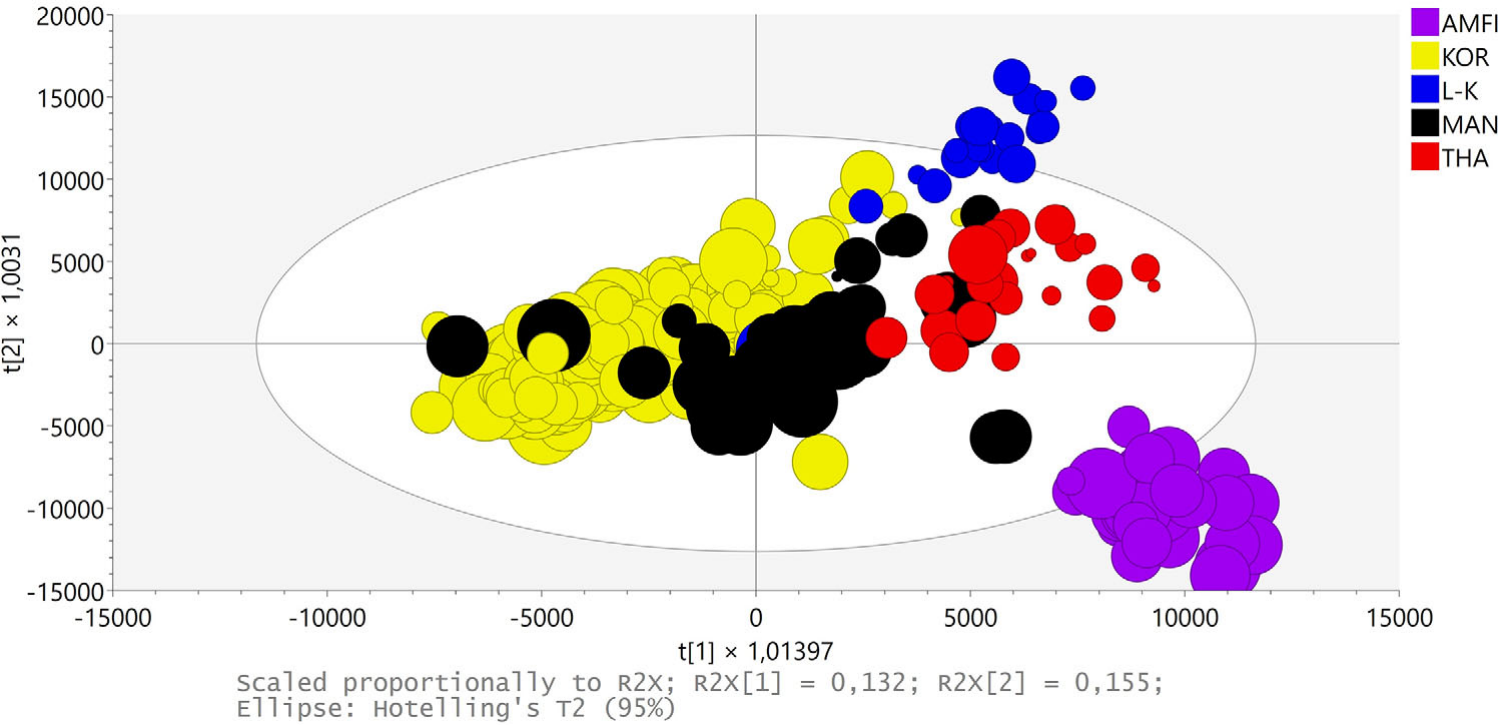
OPLS‐DA score plot of the ^1^H NMR dataset of the olive leaf extracts coloured according to their botanical origins: AMFI, KOR, L‐K, MAN and THA. For each sample, the dot size is proportional to the intensity of the oleuropein vinyl proton at *δ*_H_ 7.44, reflecting the relative abundance of this compound across the samples. The sample dot size illustrated as oleuropein variation was orthogonal to the cultivar‐related discrimination, precluding its reliability for chemotaxonomic fine‐scale classification.

Most of the OPLS‐DA discriminant variables (Table S5) belonged to triterpenic acids, particularly to oleanolic and maslinic acids. A negative correlation was observed for the resonance at *δ*_H_ 3.75, which was attributed to mannitol, indicating a relatively higher abundance of this metabolite in the cultivar located in the left quadrants. The opposite trend was observed for triterpenoids, which showed a positive correlation with t[1] and were more abundant in the bottom right quadrant. Unlike triterpenoids, mannitol is a primary metabolite involved in carbon transport and osmotic regulation in olive trees [37]. Although primary metabolites are generally not considered reliable chemotaxonomic markers, previous studies have demonstrated genotype‐dependent differences in mannitol metabolism and transport, particularly under abiotic stress conditions [37, 38]. The distinct accumulation patterns observed in our analysis suggest that mannitol levels may still reflect underlying genetic regulation across cultivars, despite their primary metabolic role. Interestingly, none of the oleuropein signals that influenced the sample distribution in the PCA was found among the important contributors of the OPLS‐DA model. In fact, when targeting one of the most diagnostic and nonoverlapping signals of oleuropein, namely, the singlet at *δ*_H_ 7.44 corresponding to the vinyl proton in Position 3, the VIP score was negligible (0.0013) with a low p(corr)[1] value of −0.26. This suggests that the inter‐cultivar variability of oleuropein was smaller than the intra‐cultivar variability in our dataset. As a result, the oleuropein‐related variance was largely projected on the orthogonal components rather than on the predictive component associated with the cultivar. To visualise oleuropein variance in the dataset, the scatter plot was modified according to the intensity of the singlet at *δ*_H_ 7.44. In Figure 5, the sample dot size is proportional to the signal intensity, reflecting the relative abundance of this compound across the olive leaf extracts. The distribution pattern showed that the oleuropein content varied continuously within and among the cultivars, without a clear correspondence with the clustering pattern generated by the OPLS‐DA model. The sample dot size representing oleuropein variation was orthogonal to the cultivar‐related discrimination; in fact, cultivars positioned on both the positive and negative sides of t[1] displayed similar values.

Oleuropein is often reported as the predominant secoiridoid in olive leaves, and its ubiquity diminishes its utility as a discriminating marker for cultivar identification. Moreover, it is one of the olive leaf metabolites most affected by variation in content due to environmental, developmental and physiological factors [39, 40]. Consequently, although oleuropein is a cornerstone of the olive leaf metabolome, its intra‐cultivar variability and inter‐cultivar overlap preclude its reliability for chemotaxonomic fine‐scale classification. This limitation is substantiated by several published metabolomic studies, where oleuropein is absent from the list of significant markers in supervised multivariate models. For instance, Difonzo et al., in their investigation on Apulian olive leaf extracts, reported that partial least squares discriminant analysis (PLS‐DA) separation was primarily driven by specific flavonoids and other discriminant metabolites rather than by the abundant oleuropein [32]. Similarly, Borghini et al. demonstrated that clustering of Tuscan cultivars was attributed to minor, structurally diverse secoiridoids (e.g., caffeoyl‐secologanoloside), phenylethanoids such as verbascoside, and flavane glycosides such as apigenin‐7‐O‐glucoside [30]. In a temporal study across phenological stages, Kabbash et al. identified triterpenoids (e.g., oleanolic and maslinic acid) and flavone glycosides as the primary contributors to PCA separation involving 12 olive leaf genotypes, highlighting the stability and discriminatory power of these compound classes over oleuropein [41]. The findings of the present study align with and reinforce this emerging consensus and establish triterpenic acids as biomarkers for Greek cultivar authentication and traceability.

To investigate whether mannitol accumulation was associated with irrigation practices, an OPLS‐DA model was developed setting, the irrigation status (irrigated vs. nonirrigated) as the *Y* variable and the full set of NMR bucket intensities as *X* variables. The resulting model did not reveal a meaningful class separation (Figure S2), indicating that irrigation did not generate a detectable metabolomic shift. Mannitol resonances were not associated with a statistically relevant discriminatory power. Despite possessing VIP > 1, the |p(corr)| values were < 0.5, even if four mannitol chemical shifts out of five reached values between 0.46 and 0.49, thus just below the threshold of statistical significance generally considered indicative of a robust covariation with the response variable. This demonstrated that mannitol levels did not systematically differ between irrigated and nonirrigated samples, suggesting that its accumulation is primarily driven by intrinsic cultivar‐dependent physiological mechanisms rather than the irrigation regime alone. Consequently, mannitol cannot be considered an index of water availability in this dataset.

### 3.3 Univariate Analysis of Data

Multivariate data analysis allowed the identification of the spectral variables responsible for the main differences among the olive leaf samples, such as oleuropein‐ and mannitol‐related resonances, and enabled us to identify chemotaxonomic markers for cultivar classification, that is, triterpenic acids, which emerged as the most important metabolites with chemosystematic value. Further examination of the 1D and 2D NMR spectra, together with the literature data and in‐house reference information, allowed the identification of compounds, major and known constituents of olive leaves, from other chemical classes, whose differences among the samples were less incisive compared to the abovementioned metabolites. Specifically, the flavones apigenin and luteolin, the flavonol quercetin and the dicarboxylic acid succinic acid were assigned from diagnostic or representative resonances. To identify subtle differences in the relative content of these metabolites among the five cultivars, a diagnostic nonoverlapping signal was selected for every compound, and ANOVA employing violin plots was performed to visualise the data.

**FIGURE 6:**
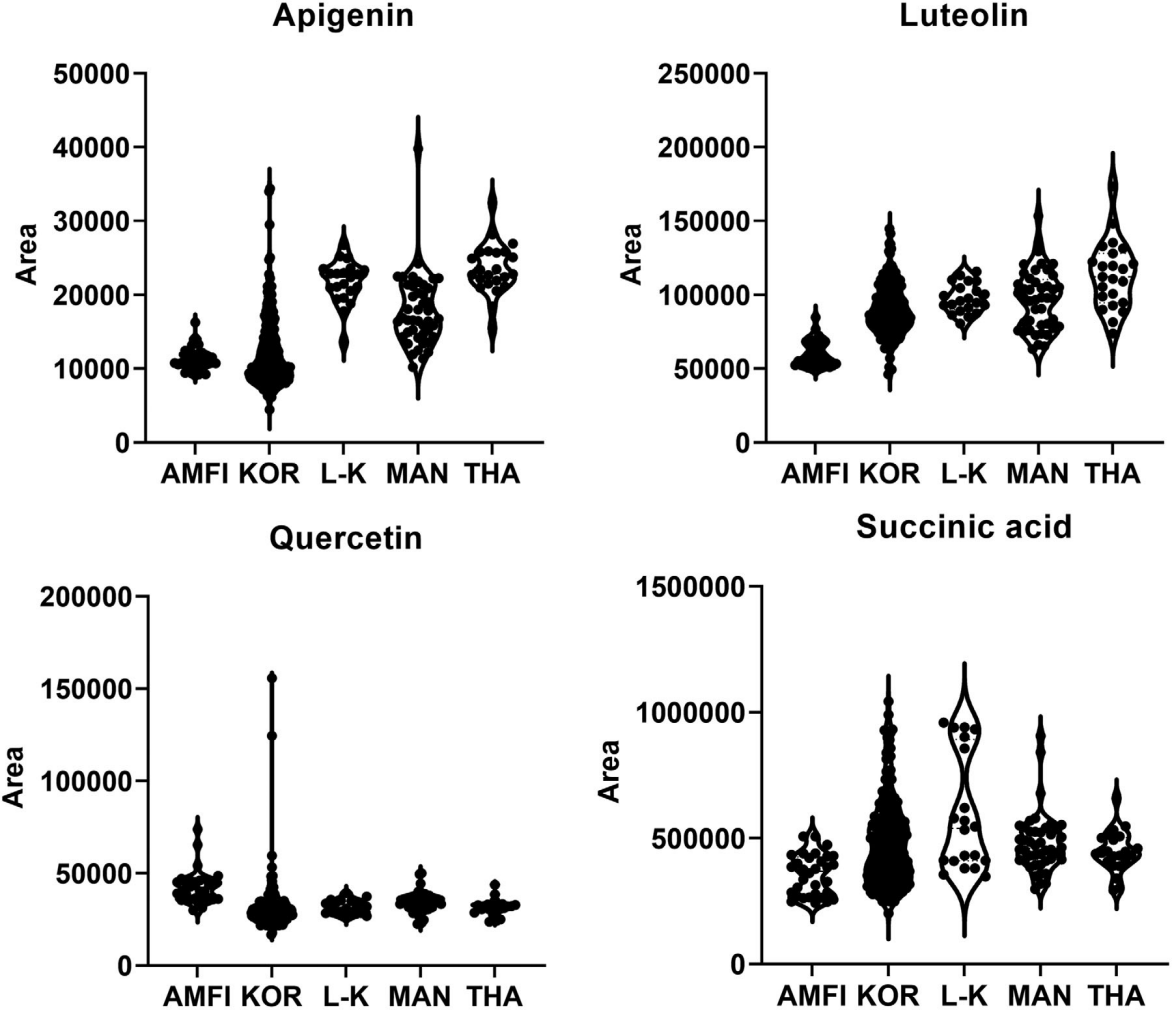
Violin plots displaying the kernel density distribution of apigenin, luteolin, quercetin and succinic acid in olive leaf extracts from five cultivars (AMFI, KOR, L‐K, MAN and THA). The varying shapes and positions of the plots highlight differences in the phytochemical profiles. The results of Tukey's HSD post hoc tests for multiple comparisons of means are presented in Table S6.

Regarding the relative signal intensities of the identified flavonoids, AMFI and L‐K cultivars displayed less intra‐cultivar variability in apigenin‐ and luteolin‐related signals compared to the other varieties (Figure 6). AMFI showed the lowest overall levels for both flavonoids, while MAN and THA showed the highest levels. Conversely, AMFI reached the highest NMR intensity values for quercetin, which was found at similar relative levels in the other cultivars, suggesting a relatively conserved quercetin content across these genotypes. Flavonoid expression in olive leaves is driven by genetic and environmental factors that modulate the activation of specific flavonoid biosynthetic pathways. Both apigenin and luteolin are produced from flavanones by flavone synthase (FNS). FNS catalyses the formation of a double bond between the carbons in Positions 2 and 3 in the C ring of flavanones. Specifically, when naringenin is produced through the phenylpropanoid‐flavonoid pathway, it can be either converted into apigenin by FNS or hydroxylated by flavanone‐3′‐hydroxylase (F3′H), generating eriodictyol, which is then converted to luteolin by FNS [42, 43]. Conversely, quercetin is produced from the flavanone eriodictyol, which is hydroxylated by flavanone‐3‐hydroxylase (F3H), forming dihydroquercetin and the latter is converted to quercetin by flavonol synthase (FLS) [43, 44]. Their roles are multiple and concern the enhancement of plant resistance to reactive oxygen species, UVB radiation and pathogen invasions [45]. Tighter regulation of the phenylpropanoid fluxes, possibly reflecting stabilised ecophysiological strategies under long‐term selection, could explain the reduced variability observed within the cultivars. Regarding flavonoid reliability as a possible marker for cultivar differentiation, the scientific literature reports contrasting results. Meirinhos et al. did not find marked differences in the phenolic profiles of 23 leaf samples from 18 Portuguese olive cultivars, finding a common flavonoid pattern [46]. On the other hand, more recent studies noted considerable variations in these phytochemicals among olive leaves from different botanical origins, listing several flavonoids, including apigenin and luteolin, together with triterpenic acids or some secoridoids, as cultivar‐discriminating factors [30, 41, 47, 48].

Succinic acid did not emerge as a cultivar discriminant in a chemotaxonomic context, showing broad and overlapping violin density plots. As a central tricarboxylic acid cycle intermediate, the level of succinic acid in olive leaves may reflect physiological and metabolic adjustments rather than purely genetic differences. Together with glucose and other organic acids, succinic acid can be metabolised by olive stressed plants for the generation of energy and carbon skeletons for the biosynthesis of metabolites in stress defence [19, 49]. Thus, variation in succinate may act as a proxy for olive leaves' metabolic state or stress response rather than defining the botanical origin.

**FIGURE 7:**
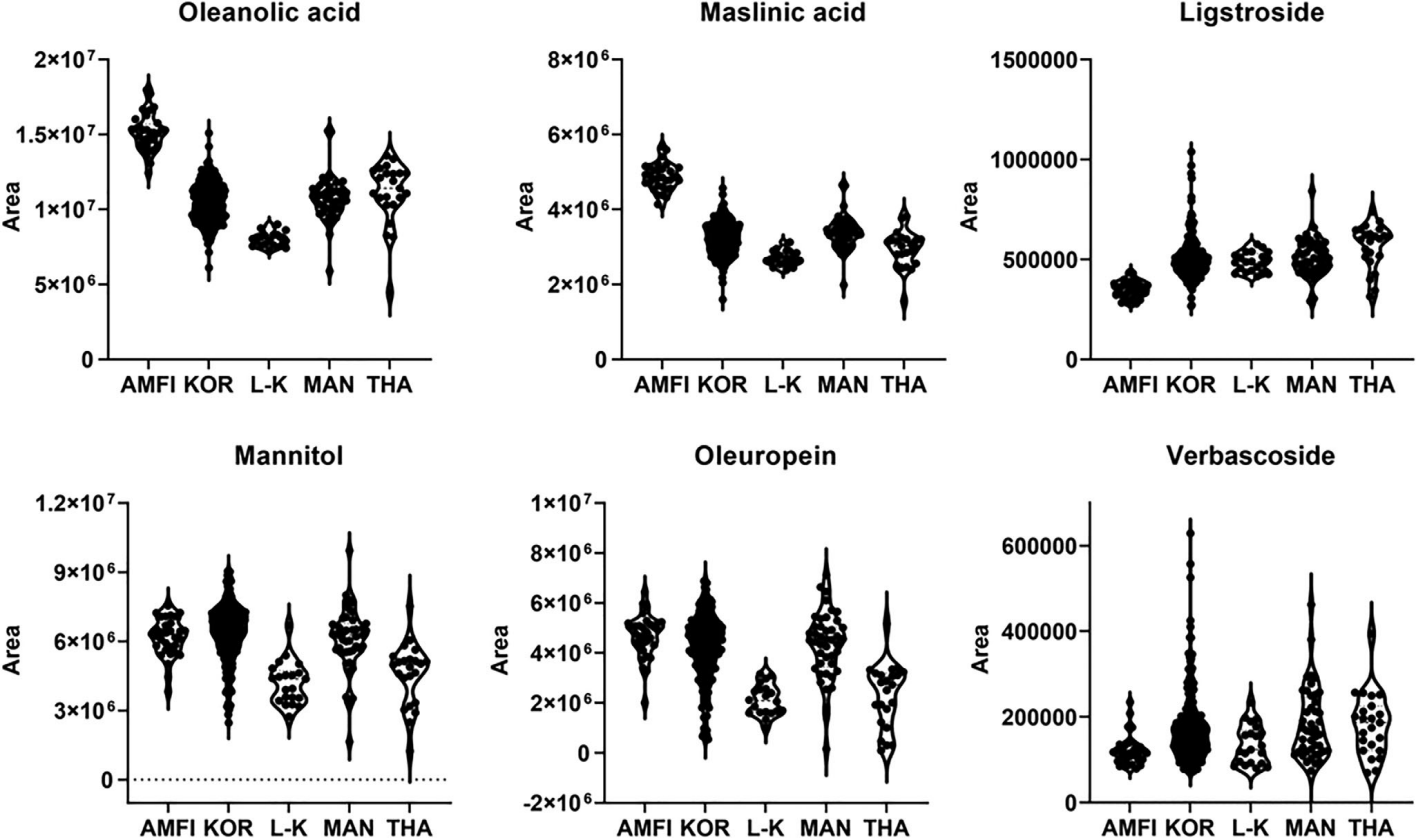
Violin plots displaying the kernel density distribution of oleanolic acid, maslinic acid, asiatic acid, mannitol oleuropein and verbascoside. The varying shapes and positions of the plots highlight significant metabolomic differences. The results of Tukey's HSD post hoc tests for multiple comparisons of means are presented in Table S6.

To evaluate the consistency of the outcomes produced from PCA and OPLS‐DA, violin plots were also employed to visualise the variations in the identified triterpenic acids, verbascoside, ligstroside, mannitol and oleuropein (Figure 7). The distribution pattern displayed by the triterpenic acids revealed cultivar‐dependent metabolomic profiles, with AMFI being the most triterpenoid‐enriched variety, consistent with the multivariate structure captured by the OPLS‐DA model. L‐K displayed the lowest relative levels for most of the investigated metabolites, with the exception of verbascoside and luteolin, whose distributions were comparable across cultivars, and apigenin, for which L‐K ranked among the most enriched cultivars, alongside MAN and THA. KOR and MAN showed close distribution trends for the majority of the analysed metabolites, confirming the partial clustering and overlap previously observed in the PCA score plot. Significant differences among the cultivars were confirmed by univariate analysis, assigning triterpenoids as cultivar‐specific biomarkers. Looking into the influence of the olive growth cycle on olive leaf triterpenoid content, a previous study found significant differences in the concentrations of these metabolites among six Spanish olive tree cultivars [50]. In agreement with our findings, they found that oleanolic and maslinic acid are the most abundant triterpenoids in olive leaves, with variations within the same cultivar being time dependent and assessing that their decrease during the selected time frames (June, August, October and December) was never more than 15.5% [50]. The strong contribution of oleanolic and maslinic acid to cultivar separation can be interpreted within the framework of genetic control of oxidosqualene cyclase (OSCs) and CYP450‐mediated oxidative tailoring steps, which are known to vary markedly among olive genotypes [31]. Recent functional genomics studies demonstrated that β‐amyrin synthase (OeBAS) and the oxidation catalyser CYP716A subfamily show cultivar‐specific allelic variants and expression patterns, resulting in different accumulation levels of triterpenoids [51]. Leaves represent a metabolically active site for biosynthesis and deposition of these compounds into the cuticle and epidermal tissues, where they contribute to resistance against UVB‐induced damage, pathogens and water loss, traits heavily shaped by the cultivar's evolutionary and agronomic history [52]. The high triterpenoid content observed in the cultivar AMFI may therefore reflect a genotype adaptation to xeric or pathogen‐rich environments.

The variation in verbascoside among the cultivars was limited and did not represent a strong discriminatory feature; however, different contents of this compound were recorded within samples belonging to the same botanical origin. Fluctuations of this phenylethanoid derivative were found to be mostly related to the availability of nutrients, particularly K and Cu, with which verbascoside exhibited negative and positive correlations, respectively [53], as well as with weather conditions since low temperatures are negatively correlated with verbascoside expression [54]. These findings could explain the different contents of verbascoside in our samples, which were collected in different geographical areas and during different periods.

Mannitol and oleuropein showed violin plots with the most intra‐cultivar heterogeneity. In particular, oleuropein displayed a very high variability, being below the detection limit in a subset of samples, while very abundant in others, reflecting the marked seasonal and cultivar‐dependent fluctuations in its accumulation. The univariate analysis corroborated that the expression of this secoiridoid cannot be related solely or predominantly to the botanical origins. Compared to oleuropein, mannitol reached a more restricted variability, and one of its resonances was retrieved as VIP in the multivariate supervised analysis, thus contributing to cultivar discrimination. Mannitol is a sugar alcohol that is synthesised as an end product of photosynthetic CO_2_ fixation in leaves and can be exported to olive fruits in response to metabolic requirements [55]. It is one of the most abundant sugars produced by olive leaves and plays several roles, especially related to stress conditions. Mannitol accumulation contributes to osmotic adjustment during stress due to salinity [37, 38], drought [56] and low temperatures [57]. Under these abiotic stress conditions, this sugar alcohol accumulates in the leaves, contributing to lowering the osmotic potential and maintaining membrane integrity and proper cellular functions. Moreover, mannitol and other polyols may act as scavengers of highly reactive hydroxyl radicals, offering a non‐enzymatic mechanism for cell protection from oxidative stress and photosystem damage [58, 59]. These different functions could explain the heterogeneity displayed by the violin plots. Despite being a primary metabolite mostly connected to the physiological state of the plant, several studies reported cultivar‐related expression of mannitol, corroborating our findings [58, 60, 61]. Although mannitol is a primary stress‐responsive metabolite and therefore not a stable chemotaxonomic marker, variations in its content among the cultivars, as highlighted by the OPLS‐DA model, may reflect differences in their ability to tolerate abiotic stresses.

The violin plot of ligstroside revealed a relatively homogeneous distribution among the cultivars, except for AMFI, which exhibited the lowest levels of this metabolite. Conversely, KOR and MAN showed slightly broader distributions and reached the highest relative concentrations. A visual comparison with the corresponding plot for oleuropein suggested a slight inverse trend between the two metabolites, which was particularly evident in AMFI. This observation may be explained by their shared biosynthetic origin; oleuropein is derived from the oxidation of the hydroxytyrosol moiety of ligstroside [62]. These accumulation patterns could therefore reflect cultivar‐specific differences in the regulation of glycosylation or esterification steps leading to the synthesis of oleuropein and ligstroside, respectively [63].

In summary, taken together, the results showed that among the five cultivars, AMFI exhibited the highest and most stable triterpenoid accumulation, revealing a possible adaptive advantage in xeric or pathogen‐rich environments and positioning it as the best source for nutraceutical and pharmacological applications targeting anti‐inflammatory, antimicrobial or anticancer activities [64, 65]. Flavonoids, specifically apigenin, luteolin and quercetin, also varied among the cultivars but contributed more moderately to class separation. MAN and THA displayed the highest relative content of these strong antioxidants, which in plants strongly act as photoprotectors and in stress responses. Therefore, these cultivars could be mainly exploited for the development of antioxidant functional food and cosmetic formulations [66]. Moreover, flavonoid‐rich olive leaf extracts constitute a promising packaging material since their radical scavenging and antimicrobial properties can enhance food preservation and reduce the use of synthetic additives [67, 68]. Oleuropein reached the highest intra‐cultivar variability, most likely due to environmentally and physiologically driven fluctuations, which makes this secoiridoid unsuitable as a chemotaxonomic marker. Despite presenting great heterogeneity, KOR and MAN showed the highest relative values of oleuropein content, as well as mannitol. Thus, these cultivars could be preferred for the extraction of these specific compounds, which can be used for the production of antidiabetic, cardioprotective and anti‐ageing formulations in the case of oleuropein [69, 70, 71], while mannitol can be used for moisturising cosmetic applications, non‐glycaemic sweetening nutraceuticals and agronomic biostimulant products [72, 73]. Mannitol, although a primary metabolite, displayed cultivar‐specific trends, reflecting different genotypic capacities for osmotic adjustment and stress‐response metabolism. L‐K was the cultivar with the lowest relative concentrations of triterpenic acids, oleuropein and mannitol, making it less appealing for industrial‐level extraction of these bioactive compounds. However, the remarkably low intra‐cultivar variability found in most of the analyses makes this metabolically stabilised genotype an interesting model for studying the genetic canalisation of specialised metabolites. This study, based on an integrated approach, positions Greek olive leaves as chemically rich, cultivar‐dependent bioresources. The metabolic signatures highlighted provide new insights for traceability, agronomic selection and breeding, and support the development of high‐value leaf‐derived products.

### 3.4 Annotation of Olive Leaf Metabolites Through LC–HRMS/MS Dereplication

To complement the NMR‐based metabolomic characterisation and to strengthen the structural assignment of key marker metabolites, LC–HRMS/MS analysis was carried out on four representative extracts selected for their divergent NMR fingerprints. The fact that MS signal intensity depends on analyte ionisation efficiency and matrix effects, combined with the lack of reference standards for the majority of annotated metabolites, means that LC–HRMS/MS was used here for dereplication rather than for quantitative comparisons across the full sample set.

Fragmentation behaviour was particularly informative for dereplication of the major chemical families detected and was compared with previously reported olive leaf and *Olea* metabolite MS/MS data. All putatively annotated metabolites have been previously reported in different *Olea europaea* plant parts [26, 27, 28, 29, 30, 31, 32]. Secoiridoids (e.g., oleoside, secologanoside, oleuropein/ligstroside derivatives) consistently produced abundant low‐mass ions in the negative mode and characteristic fragments in the positive mode, reflecting cleavage of the glycosidic bond and fragmentation of the elenolic acid moiety. For example, oleoside and secoxyloganin yielded diagnostic product ions at *m*/*z* 59.0142 and 121.0666, while oleuropein‐related metabolites showed rich fragmentation patterns that included *m*/*z* 95.0501, 101.0243, 111.0087, 139.0036/139.0400 and 149.0242, supporting the annotation of multiple isomers (Table S7).

Flavonoid glycosides were readily recognised by neutral losses corresponding to hexosyl or deoxyhexosyl units and by formation of the aglycone ions. Luteolin/kaempferol‐containing metabolites generated the diagnostic aglycone at *m*/*z* 285.0401 (negative) and 287.0550 (positive), apigenin at *m*/*z* 269.0453/271.0600 and quercetin at *m*/*z* 301.0351/303.0498, often accompanied by additional ring‐cleavage fragments such as *m*/*z* 151.0035 and 178.9984 (Table S7). Phenylpropanoid glycosides (verbascoside and calceolarioside) produced intense caffeoyl‐related ions (*m*/*z* 161.0242 in negative and 163.0390 in positive mode), which, together with higher‐mass diagnostic fragments (e.g., *m*/*z* 461.1658 for verbascoside), facilitated rapid recognition of phenylpropanoid conjugates and their isomers (Table S7).

Pentacyclic triterpenic acids (e.g., oleanolic, maslinic, corosolic and related acids) displayed stable deprotonated molecules in negative mode (e.g., *m*/*z* 455.3531 or 471.3479) and informative positive‐mode product ions (e.g., *m*/*z* 203.1794 and 189.1637), enabling reliable assignment within a class that is highly relevant to cultivar discrimination in the present NMR dataset. In parallel, hydroxy‐ and oxo‐fatty acids showed predominant deprotonated molecules with limited fragmentation, consistent with their structural simplicity, adding to the list of compounds dereplicated by LC–HRMS/MS (Table S7).

Overall, the dereplication expanded the annotated chemical space of the olive leaf extracts beyond what is directly accessible by ^1^H NMR alone, and importantly, it provided orthogonal evidence for the structural identity of metabolites underpinning cultivar discrimination. Reporting compound class‐specific fragmentation patterns increases the transparency and reproducibility of the annotation process and supports the interpretation of chemometric markers in terms of biosynthetic origin and functional relevance. Comprehensive spectrometric information (accurate mass, proposed formula, RDBeq and HRMS/MS fragments in both ionisation modes) is provided in Table S7, and a representative base‐peak LC–ESI(−/+)–HRMS chromatogram is shown in Figure S3.

## 4 Conclusions

This study provides the first large‐scale metabolomic mapping of Greek olive tree leaves by integrating untargeted ^1^H NMR profiling with LC–HRMS dereplication, enabling a comprehensive characterisation of 340 samples from the five most widespread Greek cultivars. The results demonstrated that botanical origin is the dominant source of metabolic variation, surpassing the influence of geographical, agronomic and seasonally related factors. Among the detected metabolites, pentacyclic triterpenic acids, particularly oleanolic and maslinic acid, emerged as the most robust chemotaxonomic markers driving cultivar discrimination in the MVA models. In contrast, oleuropein, despite being the most abundant secoridoid in olive leaves, exhibited pronounced intra‐cultivar variability, confirming its limited suitability as a reliable cultivar marker. Flavonoids and the polyol mannitol displayed cultivar‐related accumulation trends that likely reflect genotype‐dependent physiological responses to environmental stress. The AMFI cultivar was characterised by the highest and most stable triterpenoid accumulation, whereas MAN and THA exhibited higher flavonoid levels. KOR and MAN showed the highest oleuropein and mannitol contents, highlighting their potential as sources of these bioactive compounds. Overall, this work establishes a metabolomics‐driven framework for olive leaf chemotaxonomy and cultivar authentication and provides new insights supporting the genotype‐based valorisation of olive leaves as a sustainable source of high‐value phytochemicals.

## Author Contributions

**Mariacaterina Lianza**: writing – review and editing, data curation, software, investigation, visualisation, formal analysis, validation, writing – original draft. **Stavros Beteinakis**: writing – review and editing, investigation, validation, methodology, formal analysis, software, data curation, visualisation, writing – original draft. **Maria Halabalaki**: writing – review and editing, supervision, resources, project administration, funding acquisition, conceptualisation. **Vasileios Siderakis**: methodology, formal analysis. **Panagiotis Stathopoulos**: methodology, investigation, formal analysis. **Emmanuel Hatzakis**: writing – review and editing, supervision, validation.

## Conflicts of Interest

The authors declare no conflicts of interest.

## Ethics Statement

The authors have nothing to report.

## AI‐use Statement

No artificial intelligence tools were used in the preparation of this manuscript.

## Supporting information




**Supporting File**: ansa70094‐sup‐0001‐SuppMat.pdf.

## Data Availability

The data that support the findings of this study are available from the corresponding author upon reasonable request.
