# Supplementary material for: Metabolomic Mapping of Greek Olive Leaves by Untargeted NMR‐Based Profiling and LC–HRMS Dereplication
Source: Anal Sci Adv. 2026 Jun 30;7(2):e70094. doi: 10.1002/ansa.70094 (PMC13318240; doi:10.1002/ansa.70094)
Supplement: Supplementary file 1 — Supporting File: ansa70094‐sup‐0001‐SuppMat.pdf. [file ANSA-7-e70094-s001.pdf]

Supplementary data
for

Metabolomic mapping of Greek olive leaves by untargeted NMR-based profiling and LC-HRMS dereplication

Mariacaterina Lianza[1,#], Stavros Beteinakis[1,#], Vasileios Siderakis[1], Panagiotis Stathopoulos[1], Emmanuel Hatzakis[1,2], Maria Halabalaki[1,*]

1 Division of Pharmacognosy and Natural Products Chemistry, Department of Pharmacy, National and Kapodistrian University of Athens, Panepistimiopolis, Zografou, 15771 Athens, Greece

2 Department of Food Science and Technology, The Ohio State University, 2015 Fyffe Road Columbus, 43210 Ohio, USA

# These authors contributed equally to this work.

* Correspondence: Maria Halabalaki (mariahal@pharm.uoa.gr)

Supplementary outline

FIGURES

FIGURE S1 | PCA score scatter plots showing the 340 olive leaf samples colored according to the secondary observations considered in the multivariate analysis model (PC1 explained the 36.3% of variation, PC2 the 20.1%). (A) PCA score scatter plot coloured according to the farming method (conventional or organic). (B) PCA score scatter plot coloured according to the sampling date located in two periods of the year (January-September or February-August). (C) PCA score scatter plot coloured according to the geographical areas of collection (Crete, Dodekanisa, Dytiki Ellada, Eastern Macedonia, Epirus, Ionian Island, Peloponnese, Sterea Ellada, Thessalia). (D) PCA score scatter plot coloured according to the tree age (over eighty years old or under eighty years old). (E) PCA score scatter plot coloured according to the year of collection (2019, 2020, 2021). (F) PCA score scatter plot coloured according to the irrigation regime, distinguishing samples from trees under regular irrigation (Yes) or from non-irrigated (rainfed) trees (No). The PCA model did not highlight any outstanding correlation between the sample chemical profiles and the secondary obsarvations pointing out that the botanical origin represented the main source of variation among the olive leaves samples.

FIGURE S2 | OPLS-DA score scatter plot obtained from the 1H NMR data of the olive leaf extracts, using the irrigation regime as Y-variable. The irrigated trees are indicated with green color and the non-irrigated ones (rainfed) with blue. The model did not reveal class separation and the mannitol resonances (δ_H) were not associated with a statically relevant discriminatory power, since they showed VIP > 1 (Variable Importance in Projection), but |p(corr)| < 0.5 (δ_H 3.75 VIP= 5.39 p(corr)= -0.49, δ_H 3.82 VIP= 1.72 p(corr)= 0.14, δ_H 3.71 VIP= 5.69 p(corr)= -0.46, δ_H 3.63 VIP= 5.29 p(corr)= -0.47, δH 3.57 VIP= 4.88 p(corr)= -0.48).

FIGURE S3 | Base peak LC–ESI(−/+)–HRMS chromatograms of a representative olive leaf extract. The compounds corresponding to the main peaks are annotated; 1: oleoside; 2: secologanoside; 3: oleoside methyl ester; 4: decarboxyl elenolic acid derivative; 5: secoxyloganin; 6: phenethyl primeveroside; 7: demethyl oleuropein; 8: luteolin/kaempferol hexoside – isomer 1; 9: verbascoside – isomer 1; 10: quercetin deoxyhexoside; 11: luteolin/kaempferol hexoside – isomer 2; 12: oleuropein hexoside; 13: methyl-luteolin hexoside – isomer 1; 14: methoxyoleuropein; 15: oleuropein – isomer 1; 16: oleuropein – isomer 2; 17: oleuropein – isomer 3; 18: quercetin; 19: ligstroside – isomer 1; 20: dihydroxypalmitic acid; 21: asiatic acid; 22: glycyrrhetic acid; 23: maslinic acid; 24: corosolic acid; 25: 3-*β*-O-cis-coumaroylmaslinic acid; 26: 3-*β*-O-trans-coumaroylmaslinic acid; 27: oleanolic acid.

TABLES

TABLE S1 | Detailed metadata of the 340 olive leaf samples from five botanical origins, namely Amfissis (AMFI), Koroneiki (KOR), Manaki (MAN), Lianolia-Kerkyra (L-K) and Thasou (THA). The samples were collected across different geographical areas in Greece (Crete, Dodekanisa, Dytiki Ellada, Eastern Macedonia, Epirus, Ionian Island, Peloponnese, Sterea Ellada, Thessalia), deriving from olive trees over eighty years old or under eighty years old (tree age), cultivated either with conventional or organic farming methods, under regular irrigation or not. The collection period covered three years, from 2019 to 2021, with a sampling date located in two periods of the year (January-September or February-August). Samples are sorted based on botanical origin.

TABLE S2 | NMR data of the main compounds identified in the extracts of olive leaves. For every diagnostic signal the carbon and proton chemical shifts with the associated multiciplity, coupling constant and assignments were reported.

TABLE S3 | The table reports the results of the cross-validation analysis of variance (CV-ANOVA) performed on the OPLS-DA models developed for each olive cultivar using a one-versus-rest strategy. For each model, SS (Sum of Squares) represents the total regression and residual variance, while DF (Degrees of Freedom) indicates the number of independent parameters associated with each source of variance. MS (Mean Square) corresponds to the variance normalized by the respective degrees of freedom. The F-value expresses the ratio between the regression variance and the residual variance and is used to assess the statistical significance of the model. The associated *p*-value evaluates the probability that the observed separation occurs by chance, with lower values indicating higher model significance. SD (Standard Deviation) refers to the standard deviation of the residuals, providing an estimate of the unexplained variability. The consistently high F-values and extremely low *p*-values obtained for all cultivars confirm the statistical robustness of the OPLS-DA models and demonstrate that the discrimination based on botanical origin is not attributable to random variation.

TABLE S4 | Permutation test validation of the OPLS-DA model with 500 permutations was performed. The intercepts of the regression lines for the permuted R^2Y and Q^2 values are reported. Negative Q2 intercepts confirm the robustness and statistical validity of all models, indicating a low risk of overfitting.

TABLE S5 | List of the NMR variables identified as discriminant by the OPLS-DA model. Only variables with VIP (Variable Importance in Projection) > 1 and |p(corr)[1]| >0.5 were considered significant contributors to class separation. The VIP, and p(corr)[1] values, the corresponding NMR chemical shift with multiplicity, the coupling constant with the assignment, and the related compound were reported.

TABLE S6 | Results of Tukey's HSD post-hoc tests for multiple comparisons of means for all compounds included in the violin plots. Comparisons with a p value lower than 0.05 were considered statistically significant.

TABLE S7 | Tentatively identified compounds with UPLC-HRMS and HRMS/MS. Compounds are sorted based on their retention time (RT). Molecular formula, precursor ion, RDBeq value, fragments (above 10% relative abundance), and chemical category are also presented.

FIGURES

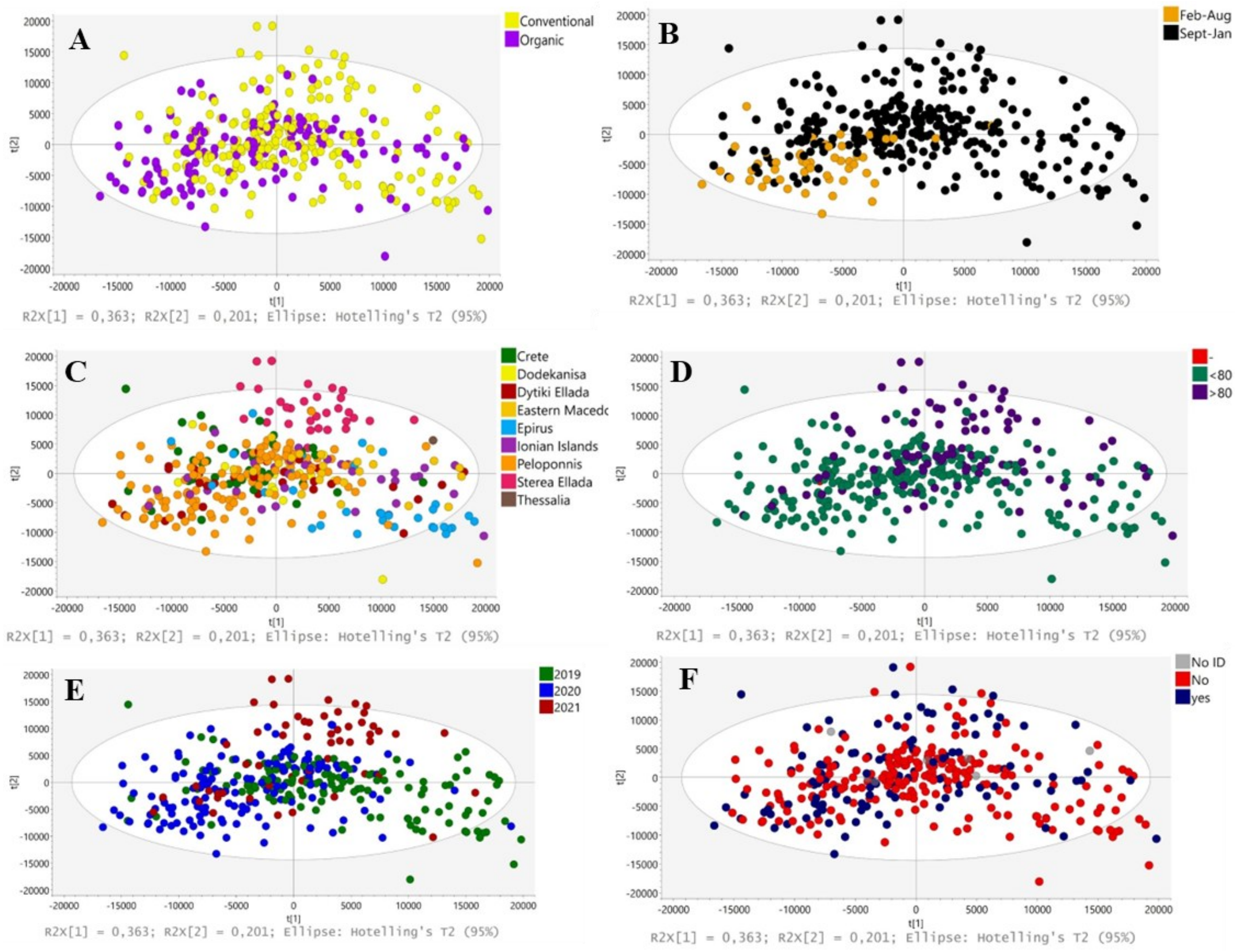

FIGURE S1 | PCA score scatter plots showing the 340 olive leaf samples colored according to the secondary observations considered in the multivariate analysis model (PC1 explained the 36.3% of variation, PC2 the 20.1%). (A) PCA score scatter plot coloured according to the farming method (conventional or organic). (B) PCA score scatter plot coloured according to the sampling date located in two periods of the year (January-September or February-August). (C) PCA score scatter plot coloured according to the geographical areas of collection (Crete, Dodekanisa, Dytiki Ellada, Eastern Macedonia, Epirus, Ionian Island, Peloponnese, Sterea Ellada, Thessalia). (D) PCA score scatter plot coloured according to the tree age (over eighty years old or under eighty years old). (E) PCA score scatter plot coloured according to the year of collection (2019, 2020, 2021). (F) PCA score scatter plot coloured according to the irrigation regime, distinguishing samples from trees under regular irrigation (Yes) or from non-irrigated (rainfed) trees (No). The PCA model did not highlight any outstanding correlation between the sample chemical profiles and the secondary obsarvations pointing out that the botanical origin represented the main source of variation among the olive leaves samples.

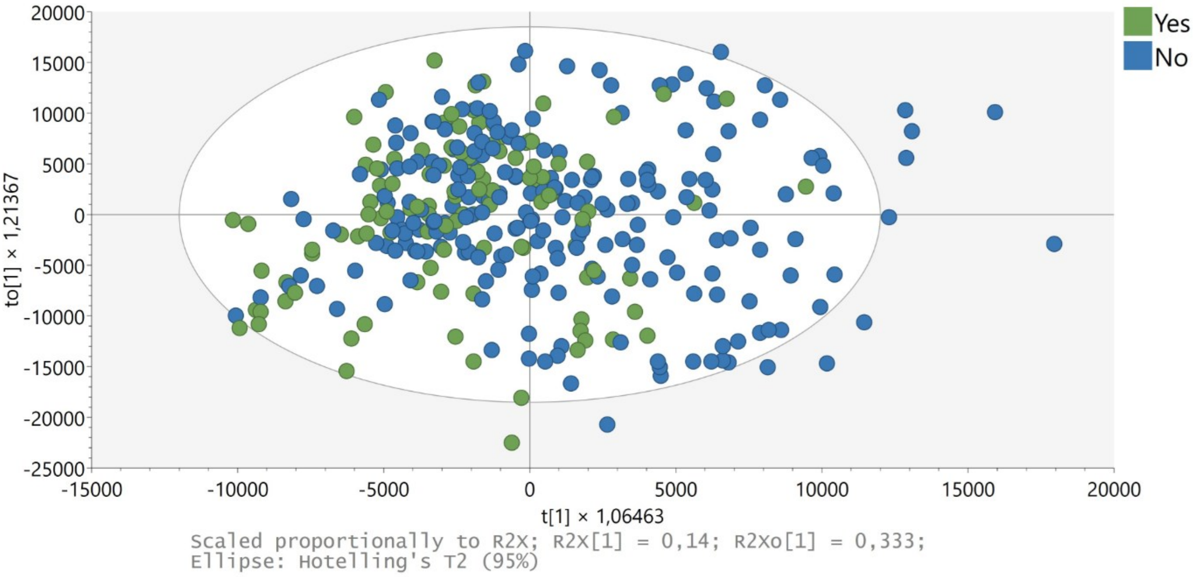

FIGURE S2 | OPLS-DA score scatter plot obtained from the ^{1}H NMR data of the olive leaf extracts, using the irrigation regime as Y-variable. The irrigated trees are indicated with green color and the non-irrigated ones (rainfed) with blue. The model did not reveal class separation and the mannitol resonances (δ_H) were not associated with a statically relevant discriminatory power, since they showed VIP > 1 (Variable Importance in Projection), but |p(corr)| < 0.5 (δ_H 3.75 VIP= 5.39 p(corr)= -0.49, δ_H 3.82 VIP= 1.72 p(corr)= 0.14, δ_H 3.71 VIP= 5.69 p(corr)= -0.46, δ_H 3.63 VIP= 5.29 p(corr)= -0.47, δ_H 3.57 VIP= 4.88 p(corr)= -0.48).

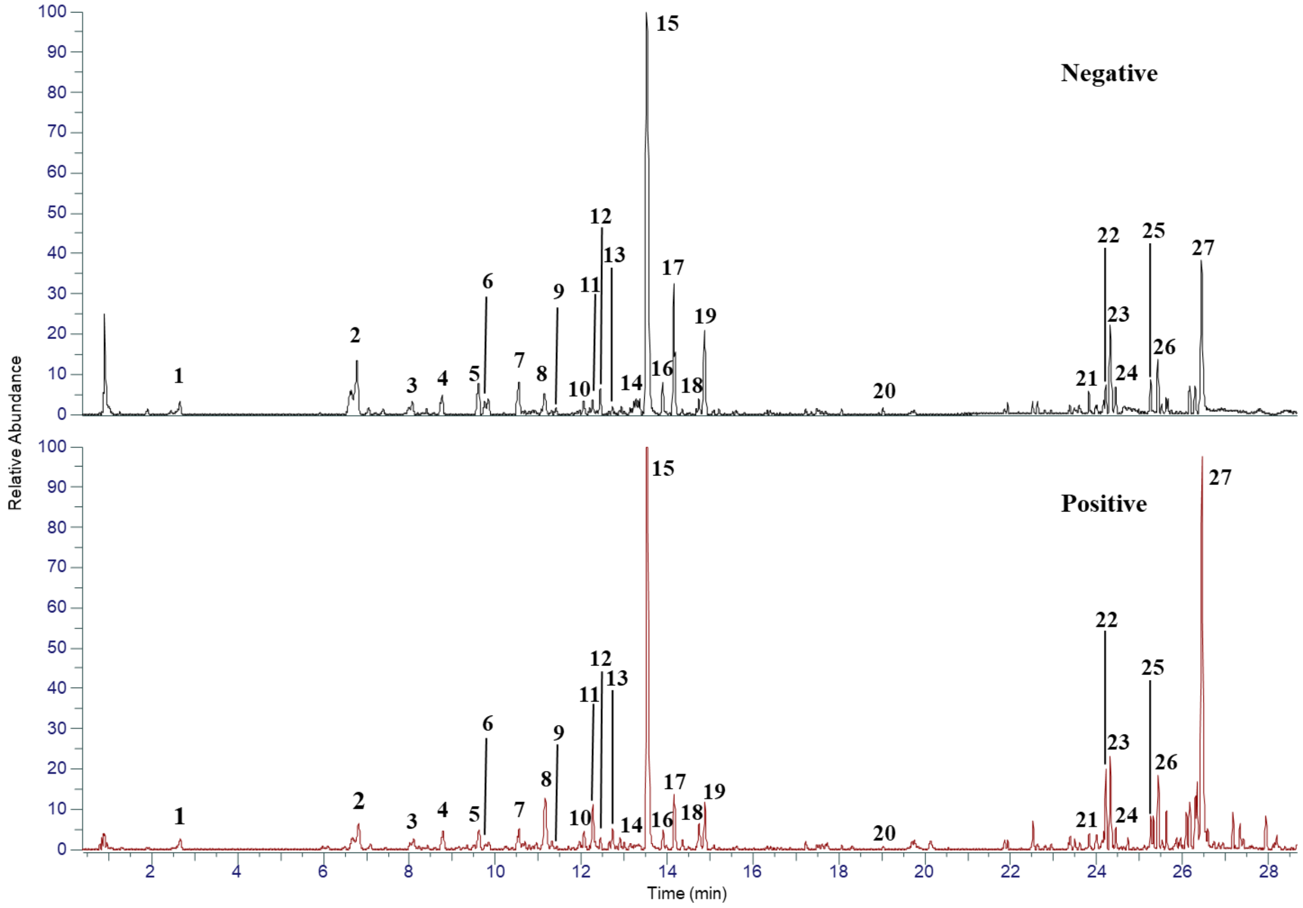

FIGURE S3 | Base peak LC–ESI(−/+)–HRMS chromatograms of a representative olive leaf extract. The compounds corresponding to the main peaks are annotated; 1: oleoside; 2: secologanoside; 3: oleoside methyl ester; 4: decarboxyl elenolic acid derivative; 5: secoxyloganin; 6: phenethyl primeveroside; 7: demethyl oleuropein; 8: luteolin/kaempferol hexoside – isomer 1; 9: verbascoside – isomer 1; 10: quercetin deoxyhexoside; 11: luteolin/kaempferol hexoside – isomer 2; 12: oleuropein hexoside; 13: methyl-luteolin hexoside – isomer 1; 14: methoxyoleuropein; 15: oleuropein – isomer 1; 16: oleuropein – isomer 2; 17: oleuropein – isomer 3; 18: quercetin; 19: ligstroside – isomer 1; 20: dihydroxypalmitic acid; 21: asiatic acid; 22: glycyrrhetic acid; 23: maslinic acid; 24: corosolic acid; 25: 3-*β*-O-cis-coumaroylmaslinic acid; 26: 3-*β*-O-trans-coumaroylmaslinic acid; 27: oleanolic acid.

TABLES

TABLE S1 │ Detailed metadata of the 340 olive leaf samples from five botanical origins, namely Amfissis (AMFI), Koroneiki (KOR), Manaki (MAN), Lianolia-Kerkyra (L-K) and Thasou (THA). The samples were collected across different geographical areas in Greece (Crete, Dodekanisa, Dytiki Ellada, Eastern Macedonia, Epirus, Ionian Island, Peloponnese, Sterea Ellada, Thessalia), deriving from olive trees over eighty years old or under eighty years old (tree age), cultivated either with conventional or organic farming methods, under regular irrigation or not. The collection period covered three years, from 2019 to 2021, with a sampling date located in two periods of the year (January-September or February-August). Samples are sorted based on botanical origin.

Sample ID	Botanical origin	Farming method	Geographical area	Geographical position	Geographical region	Collection year	Sampling date	Irrigation	Tree age
442	AMFI	Conventional	Sterea Ellada	South	Fokida	2021	Sept-Jan	Yes	>80
443	AMFI	Conventional	Sterea Ellada	south	Fokida	2021	Sept-Jan	No	>80
444	AMFI	Conventional	Sterea Ellada	south	Fokida	2021	Sept-Jan	No	>80
445	AMFI	Conventional	Sterea Ellada	south	Fokida	2021	Sept-Jan	Yes	>80
446	AMFI	Conventional	Sterea Ellada	south	Fokida	2021	Sept-Jan	No	>80
447	AMFI	Conventional	Sterea Ellada	south	Fokida	2021	Sept-Jan	Yes	>80
448	AMFI	Conventional	Sterea Ellada	south	Fokida	2021	Sept-Jan	No	>80
449	AMFI	Conventional	Sterea Ellada	south	Fokida	2021	Sept-Jan	No	>80
450	AMFI	Conventional	Sterea Ellada	south	Fokida	2021	Sept-Jan	Yes	>80
451	AMFI	Conventional	Sterea Ellada	south	Fokida	2021	Sept-Jan	Yes	>80
453	AMFI	Conventional	Sterea Ellada	south	Fokida	2021	Sept-Jan	No	>80
454	AMFI	Conventional	Sterea Ellada	south	Fokida	2021	Sept-Jan	Yes	>80
455	AMFI	Conventional	Sterea Ellada	south	Fokida	2021	Sept-Jan	Yes	>80
456	AMFI	Conventional	Sterea Ellada	south	Fokida	2021	Sept-Jan	No	>80
457	AMFI	Conventional	Sterea Ellada	south	Fokida	2021	Sept-Jan	No	>80
458	AMFI	Conventional	Sterea Ellada	south	Fokida	2021	Sept-Jan	No	>80
459	AMFI	Conventional	Sterea Ellada	south	Fokida	2021	Sept-Jan	No	>80
460	AMFI	Conventional	Sterea Ellada	south	Fokida	2021	Sept-Jan	Yes	>80
461	AMFI	Conventional	Sterea Ellada	south	Fokida	2021	Sept-Jan	Yes	>80
463	AMFI	Conventional	Sterea Ellada	south	Fokida	2021	Sept-Jan	No	>80
464	AMFI	Conventional	Sterea Ellada	south	Fokida	2021	Sept-Jan	Yes	>80
465	AMFI	Conventional	Sterea Ellada	south	Fokida	2021	Sept-Jan	Yes	>80
466	AMFI	Conventional	Sterea Ellada	south	Fokida	2021	Sept-Jan	No	>80

Sample ID	Botanical origin	Farming method	Geographical area	Geographical position	Geographical region	Collection year	Sampling date	Irrigation	Tree age
468	AMFI	Conventional	Sterea Ellada	south	Fokida	2021	Sept-Jan	Yes	>80
469	AMFI	Conventional	Sterea Ellada	south	Fokida	2021	Sept-Jan	Yes	>80
470	AMFI	Conventional	Sterea Ellada	south	Fokida	2021	Sept-Jan	Yes	>80
471	AMFI	Conventional	Sterea Ellada	south	Fokida	2021	Sept-Jan	Yes	>80
472	AMFI	Conventional	Sterea Ellada	south	Fokida	2021	Sept-Jan	No	>80
473	AMFI	Conventional	Sterea Ellada	south	Fokida	2021	Sept-Jan	Yes	>80
475	AMFI	Organic	Sterea Ellada	south	Fokida	2021	Sept-Jan	Yes	>80
476	AMFI	Conventional	Sterea Ellada	south	Fokida	2021	Sept-Jan	Yes	>80
54	AMFI	Conventional	Thessalia	north	Magnesia	2019	Sept-Jan	No	>80
361	KOR	Conventional	Epirus	north	Preveza	2020	Sept-Jan	No	<80
100	KOR	Conventional	Ionian Islands	south	Zakynthos	2019	Sept-Jan	No	<80
101	KOR	Organic	Ionian Islands	south	Zakynthos	2019	Sept-Jan	Yes	<80
102	KOR	Conventional	Ionian Islands	south	Zakynthos	2019	Sept-Jan	No	>80
103	KOR	Organic	Ionian Islands	south	Zakynthos	2019	Sept-Jan	No	>80
104	KOR	Organic	Ionian Islands	south	Zakynthos	2019	Sept-Jan	Yes	<80
105	KOR	Conventional	Ionian Islands	south	Zakynthos	2019	Sept-Jan	No	>80
150	KOR	Organic	Dodekanisa	south	Rhodes	2019	Sept-Jan	No	<80
151	KOR	Conventional	Dodekanisa	south	Rhodes	2019	Sept-Jan	No	<80
152	KOR	Conventional	Dodekanisa	south	Dodekanisa	2019	Sept-Jan	No	<80
153	KOR	Conventional	Dodekanisa	south	Rhodes	2019	Sept-Jan	No	<80
154	KOR	Conventional	Dodekanisa	south	Dodekanisa	2019	Sept-Jan	No	<80
155	KOR	Conventional	Dodekanisa	south	Dodekanisa	2019	Sept-Jan	No	<80
156	KOR	Organic	Dodekanisa	south	Dodekanisa	2019	Sept-Jan	No	<80
157	KOR	Conventional	Dodekanisa	south	Rhodes	2019	Sept-Jan	No	<80
158	KOR	Organic	Dodekanisa	south	Rhodes	2019	Sept-Jan	No	<80
159	KOR	Conventional	Dodekanisa	south	Dodekanisa	2019	Sept-Jan	No	<80
161	KOR	Organic	Dodekanisa	south	Rhodes	2019	Sept-Jan	No	<80
162	KOR	Conventional	Dodekanisa	south	Rhodes	2019	Sept-Jan	No	<80
163	KOR	Conventional	Dodekanisa	south	Rhodes	2019	Sept-Jan	No	<80
166	KOR	Organic	Dytiki Ellada	south	Aitoloakarnania	2019	Sept-Jan	No	<80
167	KOR	Conventional	Dytiki Ellada	south	Aitoloakarnania	2019	Sept-Jan	No	<80
168	KOR	Organic	Dytiki Ellada	south	Aitoloakarnania	2019	Sept-Jan	No	<80
169	KOR	Organic	Dytiki Ellada	south	Aitoloakarnania	2019	Sept-Jan	No	<80

Sample ID	Botanical origin	Farming method	Geographical area	Geographical position	Geographical region	Collection year	Sampling date	Irrigation	Tree age
171	KOR	Organic	Crete	south	Lasithi	2019	Sept-Jan	No	<80
172	KOR	Organic	Crete	south	Lasithi	2019	Sept-Jan	No	<80
173	KOR	Conventional	Crete	south	Heraklion	2019	Sept-Jan	Yes	<80
174	KOR	Organic	Crete	south	Rethymno	2019	Sept-Jan	Yes	<80
175	KOR	Conventional	Crete	south	Heraklion	2019	Sept-Jan	Yes	<80
176	KOR	Conventional	Crete	south	Heraklion	2019	Sept-Jan	Yes	<80
177	KOR	Conventional	Crete	south	Heraklion	2019	Sept-Jan	Yes	<80
178	KOR	Conventional	Crete	south	Heraklion	2019	Sept-Jan	Yes	<80
179	KOR	Conventional	Crete	south	Heraklion	2019	Sept-Jan	Yes	<80
181	KOR	Conventional	Crete	south	Heraklion	2019	Sept-Jan	Yes	<80
188	KOR	Organic	Crete	south	Heraklion	2019	Sept-Jan	No	<80
189	KOR	Conventional	Crete	south	Heraklion	2019	Sept-Jan	Yes	<80
190	KOR	Conventional	Crete	south	Heraklion	2019	Sept-Jan	Yes	<80
191	KOR	Conventional	Crete	south	Heraklion	2019	Sept-Jan	Yes	<80
192	KOR	Conventional	Crete	south	Heraklion	2019	Sept-Jan	Yes	<80
193	KOR	Conventional	Crete	south	Heraklion	2019	Sept-Jan	Yes	<80
194	KOR	Organic	Crete	south	Rethymno	2019	Sept-Jan	Yes	-
195	KOR	Conventional	Crete	south	Heraklion	2019	Sept-Jan	No	<80
196	KOR	Organic	Crete	south	Heraklion	2019	Sept-Jan	No	<80
197	KOR	Conventional	Crete	south	Heraklion	2019	Sept-Jan	No	<80
198	KOR	Conventional	Crete	south	Heraklion	2019	Sept-Jan	Yes	<80
199	KOR	Conventional	Crete	south	Heraklion	2019	Sept-Jan	Yes	<80
200	KOR	Conventional	Dytiki Ellada	south	Aitoloakarnania	2019	Sept-Jan	Yes	<80
201	KOR	Conventional	Dytiki Ellada	south	Aitoloakarnania	2019	Sept-Jan	No	<80
202	KOR	Organic	Dytiki Ellada	south	Aitoloakarnania	2019	Sept-Jan	Yes	>80
203	KOR	Conventional	Dytiki Ellada	south	Aitoloakarnania	2019	Sept-Jan	No	<80
204	KOR	Conventional	Dytiki Ellada	south	Aitoloakarnania	2019	Sept-Jan	Yes	<80
205	KOR	Conventional	Dytiki Ellada	south	Aitoloakarnania	2019	Sept-Jan	Yes	<80
206	KOR	Organic	Dytiki Ellada	south	Aitoloakarnania	2019	Sept-Jan	Yes	>80
207	KOR	Organic	Dytiki Ellada	south	Aitoloakarnania	2019	Sept-Jan	Yes	>80
208	KOR	Organic	Crete	south	Heraklion	2019	Sept-Jan	Yes	<80
209	KOR	Organic	Crete	south	Heraklion	2019	Sept-Jan	Yes	<80
210	KOR	Organic	Crete	south	Heraklion	2019	Sept-Jan	Yes	<80

Sample ID	Botanical origin	Farming method	Geographical area	Geographical position	Geographical region	Collection year	Sampling date	Irrigation	Tree age
211	KOR	Organic	Crete	south	Heraklion	2019	Sept-Jan	Yes	<80
212	KOR	Conventional	Crete	south	Heraklion	2019	Sept-Jan	Yes	<80
213	KOR	Conventional	Crete	south	Heraklion	2019	Sept-Jan	Yes	<80
214	KOR	Conventional	Crete	south	Heraklion	2019	Sept-Jan	Yes	<80
215	KOR	Conventional	Crete	south	Heraklion	2019	Sept-Jan	Yes	<80
216	KOR	Organic	Crete	south	Heraklion	2019	Sept-Jan	Yes	<80
235	KOR	Conventional	Dytiki Ellada	south	Aitoloakarnania	2020	Sept-Jan	No	<80
236	KOR	Conventional	Dytiki Ellada	south	Aitoloakarnania	2020	Sept-Jan	Yes	<80
237	KOR	Conventional	Dytiki Ellada	south	Aitoloakarnania	2020	Sept-Jan	No	<80
238	KOR	Organic	Dytiki Ellada	south	Aitoloakarnania	2020	Sept-Jan	Yes	<80
239	KOR	Conventional	Dytiki Ellada	south	Aitoloakarnania	2020	Sept-Jan	Yes	<80
240	KOR	Organic	Dytiki Ellada	south	Aitoloakarnania	2020	Sept-Jan	Yes	<80
241	KOR	Organic	Dytiki Ellada	south	Aitoloakarnania	2020	Sept-Jan	Yes	<80
242	KOR	Organic	Peloponnese	south	Messinia	2020	Sept-Jan	No	<80
243	KOR	Organic	Peloponnese	south	Messinia	2020	Sept-Jan	No	<80
244	KOR	Organic	Peloponnese	south	Messinia	2020	Sept-Jan	No	<80
245	KOR	Organic	Peloponnese	south	Messinia	2020	Sept-Jan	No	<80
246	KOR	Organic	Peloponnese	south	Messinia	2020	Sept-Jan	No	<80
247	KOR	Organic	Peloponnese	south	Messinia	2020	Sept-Jan	No	<80
248	KOR	Organic	Peloponnese	south	Messinia	2020	Sept-Jan	No	<80
249	KOR	Organic	Peloponnese	south	Messinia	2020	Sept-Jan	No	<80
250	KOR	Organic	Peloponnese	south	Messinia	2020	Sept-Jan	No	<80
251	KOR	Organic	Peloponnese	south	Messinia	2020	Sept-Jan	No	<80
252	KOR	Conventional	Peloponnese	south	Messinia	2020	Sept-Jan	No	<80
253	KOR	Conventional	Peloponnese	south	Messinia	2020	Sept-Jan	No	<80
254	KOR	Conventional	Peloponnese	south	Messinia	2020	Sept-Jan		<80
255	KOR	Conventional	Peloponnese	south	Messinia	2020	Sept-Jan	No	<80
256	KOR	Conventional	Peloponnese	south	Messinia	2020	Sept-Jan	No	<80
257	KOR	Conventional	Peloponnese	south	Messinia	2020	Sept-Jan	No	<80
258	KOR	Conventional	Peloponnese	south	Messinia	2020	Sept-Jan	No	<80
259	KOR	Conventional	Peloponnese	south	Messinia	2020	Sept-Jan	No	<80
260	KOR	Conventional	Peloponnese	south	Messinia	2020	Sept-Jan	No	<80
261	KOR	Conventional	Peloponnese	south	Messinia	2020	Sept-Jan	No	<80

Sample ID	Botanical origin	Farming method	Geographical area	Geographical position	Geographical region	Collection year	Sampling date	Irrigation	Tree age
262	KOR	Organic	Peloponnese	south	Lakonia	2020	Sept-Jan	No	<80
263	KOR	Organic	Peloponnese	south	Lakonia	2020	Sept-Jan	No	<80
264	KOR	Organic	Peloponnese	south	Lakonia	2020	Sept-Jan	No	<80
265	KOR	Organic	Peloponnese	south	Lakonia	2020	Sept-Jan	No	<80
266	KOR	Organic	Peloponnese	south	Lakonia	2020	Sept-Jan	No	<80
267	KOR	Organic	Peloponnese	south	Lakonia	2020	Sept-Jan	No	<80
268	KOR	Organic	Peloponnese	south	Lakonia	2020	Sept-Jan	No	<80
269	KOR	Organic	Peloponnese	south	Lakonia	2020	Sept-Jan	No	<80
270	KOR	Organic	Peloponnese	south	Lakonia	2020	Sept-Jan	No	<80
273	KOR	Organic	Peloponnese	south	Lakonia	2020	Sept-Jan	No	<80
274	KOR	Organic	Peloponnese	south	Lakonia	2020	Sept-Jan	No	<80
275	KOR	Organic	Peloponnese	south	Lakonia	2020	Sept-Jan	No	<80
276	KOR	Organic	Peloponnese	south	Lakonia	2020	Sept-Jan	No	<80
277	KOR	Organic	Peloponnese	south	Lakonia	2020	Sept-Jan	No	<80
278	KOR	Organic	Peloponnese	south	Lakonia	2020	Sept-Jan	No	<80
279	KOR	Organic	Peloponnese	south	Lakonia	2020	Sept-Jan	No	<80
280	KOR	Organic	Peloponnese	south	Lakonia	2020	Sept-Jan	No	<80
283	KOR	Organic	Peloponnese	south	Lakonia	2020	Sept-Jan	Yes	<80
284	KOR	Organic	Peloponnese	south	Lakonia	2020	Sept-Jan	No	<80
285	KOR	Conventional	Peloponnese	south	Lakonia	2020	Feb-Aug	No	<80
286	KOR	Conventional	Peloponnese	south	Lakonia	2020	Feb-Aug	No	<80
287	KOR	Conventional	Peloponnese	south	Lakonia	2020	Feb-Aug	No	<80
288	KOR	Conventional	Peloponnese	south	Lakonia	2020	Feb-Aug	No	<80
289	KOR	Conventional	Peloponnese	south	Lakonia	2020	Feb-Aug	No	<80
290	KOR	Conventional	Peloponnese	south	Lakonia	2020	Feb-Aug	No	<80
291	KOR	Conventional	Peloponnese	south	Lakonia	2020	Feb-Aug	No	<80
292	KOR	Conventional	Peloponnese	south	Lakonia	2020	Feb-Aug	No	<80
293	KOR	Conventional	Peloponnese	south	Lakonia	2020	Feb-Aug	No	<80
294	KOR	Conventional	Peloponnese	south	Lakonia	2020	Feb-Aug	No	<80
295	KOR	Conventional	Peloponnese	south	Lakonia	2020	Feb-Aug	No	<80
296	KOR	Conventional	Peloponnese	south	Lakonia	2020	Feb-Aug	No	<80
297	KOR	Conventional	Peloponnese	south	Lakonia	2020	Feb-Aug	No	<80
298	KOR	Conventional	Peloponnese	south	Lakonia	2020	Feb-Aug	No	<80

Sample ID	Botanical origin	Farming method	Geographical area	Geographical position	Geographical region	Collection year	Sampling date	Irrigation	Tree age
299	KOR	Conventional	Peloponnese	south	Lakonia	2020	Feb-Aug	No	<80
300	KOR	Conventional	Peloponnese	south	Lakonia	2020	Feb-Aug	No	<80
301	KOR	Conventional	Peloponnese	south	Lakonia	2020	Feb-Aug	No	<80
302	KOR	Conventional	Peloponnese	south	Lakonia	2020	Feb-Aug	No	<80
303	KOR	Conventional	Peloponnese	south	Lakonia	2020	Feb-Aug	No	<80
304	KOR	Conventional	Peloponnese	south	Lakonia	2020	Feb-Aug	No	<80
305	KOR	Organic	Peloponnese	south	Lakonia	2020	Feb-Aug	Yes	<80
306	KOR	Organic	Peloponnese	south	Lakonia	2020	Feb-Aug	Yes	<80
307	KOR	Organic	Peloponnese	south	Lakonia	2020	Feb-Aug	Yes	<80
308	KOR	Organic	Peloponnese	south	Lakonia	2020	Feb-Aug	Yes	<80
309	KOR	Organic	Peloponnese	south	Lakonia	2020	Feb-Aug	Yes	<80
310	KOR	Organic	Peloponnese	south	Lakonia	2020	Feb-Aug	Yes	<80
311	KOR	Organic	Peloponnese	south	Lakonia	2020	Feb-Aug	Yes	<80
312	KOR	Organic	Peloponnese	south	Lakonia	2020	Feb-Aug	Yes	<80
313	KOR	Organic	Peloponnese	south	Lakonia	2020	Feb-Aug	Yes	<80
314	KOR	Organic	Peloponnese	south	Lakonia	2020	Feb-Aug	Yes	<80
315	KOR	Conventional	Peloponnese	south	Lakonia	2020	Feb-Aug	Yes	<80
316	KOR	Conventional	Peloponnese	south	Lakonia	2020	Feb-Aug	Yes	<80
317	KOR	Conventional	Peloponnese	south	Lakonia	2020	Feb-Aug	Yes	<80
318	KOR	Conventional	Peloponnese	south	Lakonia	2020	Feb-Aug	Yes	<80
319	KOR	Conventional	Peloponnese	south	Lakonia	2020	Feb-Aug	Yes	<80
320	KOR	Conventional	Peloponnese	south	Lakonia	2020	Feb-Aug	Yes	<80
321	KOR	Conventional	Peloponnese	south	Lakonia	2020	Feb-Aug	Yes	<80
322	KOR	Conventional	Peloponnese	south	Lakonia	2020	Feb-Aug	Yes	<80
323	KOR	Conventional	Peloponnese	south	Lakonia	2020	Feb-Aug	Yes	<80
324	KOR	Conventional	Peloponnese	south	Lakonia	2020	Feb-Aug	Yes	<80
325	KOR	Organic	Peloponnese	south	Messinia	2020	Sept-Jan	No	<80
326	KOR	Organic	Peloponnese	south	Messinia	2020	Feb-Aug	No	<80
327	KOR	Organic	Peloponnese	south	Messinia	2020	Feb-Aug	No	<80
328	KOR	Organic	Peloponnese	south	Messinia	2020	Feb-Aug	No	<80
329	KOR	Organic	Peloponnese	south	Messinia	2020	Feb-Aug	No	<80
330	KOR	Organic	Peloponnese	south	Messinia	2020	Feb-Aug	No	<80
331	KOR	Organic	Peloponnese	south	Messinia	2020	Feb-Aug	No	<80

Sample ID	Botanical origin	Farming method	Geographical area	Geographical position	Geographical region	Collection year	Sampling date	Irrigation	Tree age
332	KOR	Organic	Peloponnese	south	Messinia	2020	Feb-Aug	No	<80
333	KOR	Organic	Peloponnese	south	Messinia	2020	Feb-Aug	No	<80
334	KOR	Organic	Peloponnese	south	Messinia	2020	Feb-Aug	No	<80
338	KOR	Organic	Crete	south	Lasithi	2020	Sept-Jan	No	>80
339	KOR	Organic	Crete	south	Lasithi	2020	Sept-Jan	Yes	>80
340	KOR	Organic	Crete	south	Lasithi	2020	Sept-Jan	Yes	>80
341	KOR	Organic	Crete	south	Lasithi	2020	Sept-Jan	No	>80
342	KOR	Conventional	Epirus	north	Thesprotia	2020	Sept-Jan	No	<80
343	KOR	Conventional	Epirus	north	Thesprotia	2020	Sept-Jan	No	<80
344	KOR	Conventional	Epirus	north	Thesprotia	2020	Sept-Jan	No	<80
345	KOR	Conventional	Epirus	north	Thesprotia	2020	Sept-Jan	No	<80
346	KOR	Conventional	Epirus	north	Thesprotia	2020	Sept-Jan	No	<80
348	KOR	Conventional	Epirus	north	Thesprotia	2020	Sept-Jan	No	<80
350	KOR	Conventional	Epirus	north	Thesprotia	2020	Sept-Jan	No	<80
354	KOR	Conventional	Epirus	north	Thesprotia	2020	Sept-Jan	No	<80
355	KOR	Organic	Crete	south	Heraklion	2020	Sept-Jan	yes	<80
356	KOR	Organic	Crete	south	Heraklion	2020	Sept-Jan	yes	<80
357	KOR	Organic	Crete	south	Heraklion	2020	Sept-Jan	yes	<80
358	KOR	Organic	Dytiki Ellada	south	Aitoloakarnania	2020	Sept-Jan	No	<80
359	KOR	Organic	Dytiki Ellada	south	Aitoloakarnania	2020	Sept-Jan	No	<80
360	KOR	Organic	Dytiki Ellada	south	Aitoloakarnania	2020	Sept-Jan	No	<80
368	KOR	Conventional	Epirus	north	Preveza	2020	Sept-Jan	No	<80
383	KOR	Organic	Crete	south	Heraklion	2020	Sept-Jan	yes	<80
384	KOR	Organic	Crete	south	Heraklion	2020	Sept-Jan	yes	<80
417	KOR	Organic	Crete	south	Heraklion	2020	Sept-Jan	yes	<80
419	KOR	Organic	Crete	south	Heraklion	2020	Sept-Jan	yes	<80
421	KOR	Organic	Ionian Islands	south	Zakynthos	2021	Sept-Jan	No	>80
422	KOR	Conventional	Ionian Islands	south	Zakynthos	2021	Sept-Jan	No	>80
423	KOR	Organic	Ionian Islands	south	Zakynthos	2021	Sept-Jan	No	>80
424	KOR	Organic	Ionian Islands	south	Zakynthos	2021	Sept-Jan	Yes	>80
426	KOR	Organic	Ionian Islands	south	Zakynthos	2021	Sept-Jan	No	>80
428	KOR	Organic	Ionian Islands	south	Zakynthos	2021	Sept-Jan	Yes	>80
429	KOR	Conventional	Ionian Islands	south	Zakynthos	2021	Sept-Jan	Yes	>80

Sample ID	Botanical origin	Farming method	Geographical area	Geographical position	Geographical region	Collection year	Sampling date	Irrigation	Tree age
430	KOR	Organic	Ionian Islands	south	Zakynthos	2021	Sept-Jan	Yes	>80
434	KOR	Organic	Ionian Islands	south	Zakynthos	2021	Sept-Jan	No	>80
436	KOR	Conventional	Ionian Islands	south	Zakynthos	2021	Sept-Jan	Yes	>80
437	KOR	Organic	Ionian Islands	south	Zakynthos	2021	Sept-Jan	No	>80
439	KOR	Conventional	Ionian Islands	south	Zakynthos	2021	Sept-Jan	Yes	>80
440	KOR	Conventional	Ionian Islands	south	Zakynthos	2021	Sept-Jan	Yes	>80
478	KOR	Organic	Dytiki Ellada	south	Aitoloakarnania	2021	Sept-Jan	Yes	<80
479	KOR	Organic	Dytiki Ellada	south	Aitoloakarnania	2021	Sept-Jan	No	<80
481	KOR	Conventional	Dytiki Ellada	south	Aitoloakarnania	2021	Sept-Jan	Yes	<80
483	KOR	Organic	Dytiki Ellada	south	Aitoloakarnania	2021	Sept-Jan	Yes	<80
485	KOR	Conventional	Dytiki Ellada	south	Aitoloakarnania	2021	Sept-Jan	No	<80
487	KOR	Conventional	Dytiki Ellada	south	Aitoloakarnania	2021	Sept-Jan	No	<80
488	KOR	Conventional	Dytiki Ellada	south	Aitoloakarnania	2021	Sept-Jan	No	<80
489	KOR	Conventional	Dytiki Ellada	south	Aitoloakarnania	2021	Sept-Jan	No	<80
490	KOR	Organic	Dytiki Ellada	south	Aitoloakarnania	2021	Sept-Jan	No	<80
492	KOR	Conventional	Epirus	north	Arta	2021	Sept-Jan	No	<80
493	KOR	Conventional	Epirus	north	Arta	2021	Sept-Jan	No	<80
497	KOR	Conventional	Epirus	north	Arta	2021	Feb-Aug	No	<80
56	KOR	Organic	Dytiki Ellada	south	Aitoloakarnania	2019	Sept-Jan	Yes	<80
84	KOR	Organic	Ionian Islands	south	Zakynthos	2019	Sept-Jan	No	>80
85	KOR	Conventional	Ionian Islands	south	Zakynthos	2019	Sept-Jan	No	>80
86	KOR	Organic	Ionian Islands	south	Zakynthos	2019	Sept-Jan	No	<80
87	KOR	Conventional	Ionian Islands	south	Zakynthos	2019	Sept-Jan	No	<80
90	KOR	Organic	Ionian Islands	south	Zakynthos	2019	Sept-Jan	Yes	>80
91	KOR	Organic	Ionian Islands	south	Zakynthos	2019	Sept-Jan	Yes	>80
92	KOR	Conventional	Ionian Islands	south	Zakynthos	2019	Sept-Jan	No	<80
93	KOR	Organic	Ionian Islands	south	Zakynthos	2019	Sept-Jan	No	<80
95	KOR	Organic	Ionian Islands	south	Zakynthos	2019	Sept-Jan	Yes	<80
96	KOR	Organic	Ionian Islands	south	Zakynthos	2019	Sept-Jan	Yes	>80
98	KOR	Conventional	Ionian Islands	south	Zakynthos	2019	Sept-Jan	No	>80
99	KOR	Conventional	Ionian Islands	south	Zakynthos	2019	Sept-Jan	No	<80
129	L-K	Conventional	Ionian Islands	south	Kerkyra	2019	Sept-Jan	No	>80
164	L-K	Conventional	Epirus	north	Preveza	2019	Sept-Jan	No	<80

Sample ID	Botanical origin	Farming method	Geographical area	Geographical position	Geographical region	Collection year	Sampling date	Irrigation	Tree age
217	L-K	Conventional	Epirus	north	Preveza	2019	Sept-Jan	No	<80
218	L-K	Conventional	Epirus	north	Preveza	2019	Sept-Jan	No	<80
219	L-K	Conventional	Epirus	north	Preveza	2019	Sept-Jan	No	<80
220	L-K	Conventional	Epirus	north	Preveza	2019	Sept-Jan	No	<80
221	L-K	Conventional	Epirus	north	Preveza	2019	Sept-Jan	No	<80
222	L-K	Conventional	Epirus	north	Preveza	2019	Sept-Jan	No	<80
223	L-K	Conventional	Epirus	north	Preveza	2019	Sept-Jan	No	<80
224	L-K	Conventional	Epirus	north	Preveza	2019	Sept-Jan	No	<80
225	L-K	Conventional	Epirus	north	Preveza	2019	Sept-Jan	No	<80
226	L-K	Conventional	Epirus	north	Preveza	2019	Sept-Jan	No	<80
227	L-K	Conventional	Epirus	north	Preveza	2019	Sept-Jan	No	<80
228	L-K	Conventional	Epirus	north	Preveza	2019	Sept-Jan	No	<80
229	L-K	Conventional	Epirus	north	Preveza	2019	Sept-Jan	No	<80
230	L-K	Conventional	Epirus	north	Preveza	2019	Sept-Jan	No	<80
231	L-K	Conventional	Epirus	north	Preveza	2019	Sept-Jan	No	<80
232	L-K	Conventional	Epirus	north	Preveza	2019	Sept-Jan	No	<80
233	L-K	Organic	Epirus	north	Preveza	2020	Sept-Jan	No	<80
234	L-K	Conventional	Epirus	north	Preveza	2020	Sept-Jan	No	<80
83	L-K	Organic	Ionian Islands	south	Kefalonia	2019	Sept-Jan	No	>80
106	MAN	Conventional	Peloponnese	south	Argolida	2019	Sept-Jan	No	<80
107	MAN	Conventional	Peloponnese	south	Argolida	2019	Sept-Jan		<80
108	MAN	Organic	Peloponnese	south	Argolida	2019	Sept-Jan	No	>80
109	MAN	Conventional	Peloponnese	south	Argolida	2019	Sept-Jan		>80
110	MAN	Conventional	Peloponnese	south	Argolida	2019	Sept-Jan		<80
111	MAN	Conventional	Peloponnese	south	Argolida	2019	Sept-Jan	No	>80
112	MAN	Conventional	Peloponnese	south	Argolida	2019	Sept-Jan		<80
113	MAN	Conventional	Peloponnese	south	Argolida	2019	Sept-Jan	No	>80
114	MAN	Conventional	Peloponnese	south	Argolida	2019	Sept-Jan	No	<80
115	MAN	Conventional	Peloponnese	south	Argolida	2019	Sept-Jan		<80
116	MAN	Conventional	Peloponnese	south	Argolida	2019	Sept-Jan	No	<80
117	MAN	Conventional	Peloponnese	south	Argolida	2019	Sept-Jan	No	>80
118	MAN	Conventional	Peloponnese	south	Argolida	2019	Sept-Jan	No	>80
119	MAN	Conventional	Peloponnese	south	Argolida	2019	Sept-Jan	No	<80

Sample ID	Botanical origin	Farming method	Geographical area	Geographical position	Geographical region	Collection year	Sampling date	Irrigation	Tree age
120	MAN	Conventional	Peloponnese	south	Argolida	2019	Sept-Jan	No	>80
121	MAN	Conventional	Peloponnese	south	Argolida	2019	Sept-Jan		>80
122	MAN	Conventional	Peloponnese	south	Argolida	2019	Sept-Jan	No	<80
123	MAN	Organic	Peloponnese	south	Argolida	2019	Sept-Jan	No	>80
124	MAN	Conventional	Peloponnese	south	Argolida	2019	Sept-Jan	No	>80
125	MAN	Conventional	Peloponnese	south	Argolida	2019	Sept-Jan	No	>80
21	MAN	Organic	Peloponnese	south	Argolida	2019	Sept-Jan	Yes	<80
30	MAN	Organic	Peloponnese	south	Argolida	2019	Sept-Jan	Yes	<80
34	MAN	Organic	Peloponnese	south	Argolida	2019	Sept-Jan	Yes	<80
387	MAN	Organic	Peloponnese	south	Argolida	2020	Sept-Jan	No	<80
389	MAN	Organic	Peloponnese	south	Argolida	2020	Sept-Jan	No	<80
390	MAN	Conventional	Peloponnese	south	Argolida	2020	Sept-Jan	No	>80
391	MAN	Organic	Peloponnese	south	Argolida	2020	Sept-Jan	No	>80
392	MAN	Conventional	Peloponnese	south	Argolida	2020	Sept-Jan	No	>80
393	MAN	Organic	Peloponnese	south	Argolida	2020	Sept-Jan	No	<80
396	MAN	Organic	Peloponnese	south	Argolida	2020	Sept-Jan	No	<80
400	MAN	Conventional	Peloponnese	south	Argolida	2020	Sept-Jan	No	<80
402	MAN	Conventional	Peloponnese	south	Argolida	2020	Sept-Jan	No	>80
403	MAN	Organic	Peloponnese	south	Argolida	2020	Sept-Jan	No	<80
404	MAN	Conventional	Peloponnese	south	Argolida	2020	Sept-Jan	No	<80
405	MAN	Organic	Peloponnese	south	Argolida	2020	Sept-Jan	No	<80
406	MAN	Conventional	Peloponnese	south	Argolida	2020	Sept-Jan	No	>80
407	MAN	Organic	Peloponnese	south	Argolida	2020	Sept-Jan	No	<80
409	MAN	Conventional	Peloponnese	south	Argolida	2020	Sept-Jan	No	>80
410	MAN	Conventional	Peloponnese	south	Argolida	2020	Sept-Jan	No	<80
411	MAN	Conventional	Peloponnese	south	Argolida	2020	Sept-Jan	No	<80
412	MAN	Conventional	Peloponnese	south	Argolida	2020	Sept-Jan	No	>80
413	MAN	Organic	Peloponnese	south	Argolida	2020	Sept-Jan	No	<80
414	MAN	Conventional	Peloponnese	south	Argolida	2020	Sept-Jan	No	<80
61	MAN	Conventional	Dodekanisa	south	Rhodes	2019	Sept-Jan	No	<80
132	THA	Organic	Eastern Macedonia	north	Kavala	2019	Sept-Jan	No	>80
133	THA	Organic	Eastern Macedonia	north	Kavala	2019	Sept-Jan	No	>80
134	THA	Organic	Eastern Macedonia	north	Kavala	2019	Sept-Jan	No	>80

Sample ID	Botanical origin	Farming method	Geographical area	Geographical position	Geographical region	Collection year	Sampling date	Irrigation	Tree age
135	THA	Conventional	Eastern Macedonia	north	Kavala	2019	Sept-Jan	No	>80
137	THA	Organic	Eastern Macedonia	north	Kavala	2019	Sept-Jan	No	>80
139	THA	Conventional	Eastern Macedonia	north	Kavala	2019	Sept-Jan	No	>80
141	THA	Conventional	Eastern Macedonia	north	Kavala	2019	Sept-Jan	No	>80
142	THA	Conventional	Eastern Macedonia	north	Kavala	2019	Sept-Jan	Yes	<80
144	THA	Conventional	Eastern Macedonia	north	Kavala	2019	Sept-Jan	No	<80
145	THA	Conventional	Eastern Macedonia	north	Kavala	2019	Sept-Jan		<80
146	THA	Organic	Eastern Macedonia	north	Kavala	2019	Sept-Jan	No	>80
147	THA	Conventional	Eastern Macedonia	north	Kavala	2019	Sept-Jan	No	<80
148	THA	Conventional	Eastern Macedonia	north	Kavala	2019	Sept-Jan	No	<80
149	THA	Conventional	Eastern Macedonia	north	Kavala	2019	Sept-Jan	No	<80
18	THA	Organic	Eastern Macedonia	north	Kavala	2019	Sept-Jan	Yes	>80
31	THA	Organic	Eastern Macedonia	north	Kavala	2019	Sept-Jan	Yes	>80
36	THA	Organic	Eastern Macedonia	north	Kavala	2019	Sept-Jan	Yes	>80
374	THA	Conventional	Eastern Macedonia	north	Kavala	2020	Sept-Jan	Yes	>80
377	THA	Conventional	Eastern Macedonia	north	Kavala	2020	Sept-Jan	No	>80
378	THA	Conventional	Eastern Macedonia	north	Kavala	2020	Sept-Jan	Yes	<80
379	THA	Conventional	Eastern Macedonia	north	Kavala	2020	Sept-Jan	No	<80
380	THA	Organic	Eastern Macedonia	north	Kavala	2020	Sept-Jan	Yes	>80
381	THA	Organic	Eastern Macedonia	north	Kavala	2020	Sept-Jan	Yes	>80

TABLE S2 | NMR data of the main compounds identified in the extracts of olive leaves. Only diagnostic peaks are presented. For every diagnostic signal the carbon and proton chemical shifts with the associated multiplicity, coupling constant and assignments were reported.

Compound	δ_H (multipliticy, *J* in Hz, assignment)	δ_C (assignment)
Oleuropein	7.44 (s, H-3), 6.63 (d, *J* = 8.0 Hz, H-7´), 6.60 (d, *J* = 2.0 Hz, H-4´), 6.49 (dd, *J* = 8.0/2.0 Hz, H-8´), 6.02 (q, *J* = 7.1 Hz, H-8), 5.84 (s, H-1), 4.14 (dt, *J* = 10.8, 7.1 Hz, H-1´a), 4.04 (dt, *J* = 10.8, 7.1 Hz, H-1´b), 3.91 (dd, *J* = 9.0/4.5 Hz, H-5), 3.83-3.28 (m, H-2´´-6´´), 3.65 (s, 11-COO$\underline{CH_3}$), 2.70 (t, *J* = 7.1 Hz, H-2´), 2.64 (dd, *J* = 14.1/4.5 Hz, H-6a), 2.38 (dd, *J* = 14.1/9.0 Hz, H-6b), 1.60 (d *J* = 7.1 Hz, H-10)	153.7 (C-3), 123.8 (C-8), 120.0 (C-8´), 115.7 (C-4´), 115.1 (C-7´), 93.9 (C-1), 65.3 (C-1´), 50.0 (11-COO$\underline{CH_3}$), 39.9 (C-6), 34.0 (C-2´), 30.5 (C-5), 12.2 (C-10)
Verbascoside	7.53 (d, *J* = 15.9 Hz, H-7´´´), 6.99 (d, *J* = 2.0 Hz, H-2´´´), 6.89 (dd, *J* = 8.2/2.0 Hz, H6´´´), 6.72 (d, *J* = 8.2 Hz, H-5´´´), 6.21 (d, *J* = 15.9 Hz, H-8´´´), 5.12 (d, *J* = 1.7 Hz, H-1´´), 4.32 (d, *J* = 8.0 Hz, H-1´), 1.03 (d, *J* = 6.2 Hz, H-6´´)	146.6 (C-7´´´), 113.9 (C-2´´´), 121.7 (C-6´´´), 115.1 (C-5´´´), 113.3 (C-8´´´), 101.7 (C-1´´), 102.9 (C-1´), 17.0 (C-6´´)
Ligstroside	6.99 (d, *J* = 8.6 Hz, H-4´/8´), 6.65 (d, *J* = 8.6 Hz, H-5´/7´),	129.7 (C-4´/8´), 115.0 (C-5´/7´)
Oleanolic acid	5.18 (t, *J* = 3.5 Hz, H-12), 3.08 (m, H-3), 2.79 (dd, *J* =4.1/14.3 Hz, H-18), 1.09 (s, H-27), 0.91 (s, H-23), 0.88 (2s, H-25, H-30), 0.84 (s, H-29), 0.76(s, H-26), 0.71 (s, H-24)	122.1 (C-12), 78.4 (C-3), 41.4 (C-18), 32.1 (C-29), 27.5 (C-23), 25.1 (C-27), 22.6 (C-30), 16.4 (C-26), 14.9 (C-24), 14.6 (C-25)
Maslinic acid	5.18 (t, *J* = 3.5 Hz, H-12), 2.84 (d, *J* = 9.6 Hz, H-3), 2.80 (m, H-18), 1.10 (s, H-27), 0.95 (s, H-23), 0.94 (s, H-24), 0.88 (s, H-30), 0.84 (s, H-29), 0.76 (s, H-26), 0.75 (s, H-25)	122.6 (C-12), 83.2 (C-3), 41.4 (C-18), 32.1 (C-29), 28.0 (C-23), 25.1 (C-27), 22.6 (C-30), 16.4 (C-26), 16.2 (C-25), 15.8 (C-24),
Luteolin	7.32 (dd, *J*= 9.0, 2.2 Hz, H-6´), 7.32 (d, *J*= 2.2 Hz, H-2´), 6.85 (d, *J*=9.0 Hz, H-5´), 6.48 (s, H-3), 6.38 (d, *J*= 2.1 Hz, H-8), 6.15 (d, *J*= 2.1 Hz, H-6)	119.0 (C-6'), 115.5 (C-5'), 112.9 (C-2'), 102.5 (C-3), 98.8 (C-6), 93.6 (C-8)
Apigenin	7.79 (d, *J*= 8.9, H-2´/6´), 6.53 (s, H-3)	128.0 (C-2´/6´), 102.5 (C-3)
Quercetin	7.67 (d, *J* = 2.2 Hz, H-2´), 6.83 (d, *J* = 8.5 Hz, H-5´), 6.33 (d, *J* = 2.0 Hz, H-8), 6.12 (d, *J* = 2.0 Hz, H-6)	114.80 (C-5´), 114.7 (C-2´), 97.9 (C-6), 93.2 (C-8)
Succinic acid	2.46 (s)	30.3
D- Mannitol	3.82 (dd, *J*= 11.9/2.0 Hz, H-1/6), 3.71 (d, *J*= 7.9, H-3/4), 3.63 (ddd, *J*= 7.9/6.2/2.0 Hz, H-1/6), 3.61 (dd, *J*= 11.9/6.2 Hz, H-1/6)	71.8 (C-2/5), 70.2 (C-3/4), 61.3 (C-1/6)
α-Glucose	5.04 (d, *J*= 3.7 Hz, H-1)	92.6 (C-1)

Compound	δ_H (multiplitiicy, *J* in Hz, assignment)	δ_C (assignment)
β-Glucose	4.41 (d, *J*= 7.8 Hz, H-1)	97.0 (C-1)

**TABLE S3 | ** The table reports the results of the cross-validation analysis of variance (CV-ANOVA) performed on the OPLS-DA models developed for each olive cultivar using a one-versus-rest strategy. For each model, SS (Sum of Squares) represents the total regression and residual variance, while DF (Degrees of Freedom) indicates the number of independent parameters associated with each source of variance. MS (Mean Square) corresponds to the variance normalized by the respective degrees of freedom. The F-value expresses the ratio between the regression variance and the residual variance and is used to assess the statistical significance of the model. The associated *p*-value evaluates the probability that the observed separation occurs by chance, with lower values indicating higher model significance. SD (Standard Deviation) refers to the standard deviation of the residuals, providing an estimate of the unexplained variability. The consistently high F-values and extremely low *p*-values obtained for all cultivars confirm the statistical robustness of the OPLS-DA models and demonstrate that the discrimination based on botanical origin is not attributable to random variation.

	SS	DF	MS	F	p	SD
**Botanical Origin AMFI**						
**Total corr.**	339	339	1	--	--	1
**Regression**	289.697	20	14.4849	93.7204	0	3.8059
**Residual**	49.3027	319	0.154554	--	--	0.393133
**Botanical Origin KOR**						
**Total corr.**	339	339	1	--	--	1
**Regression**	246.551	20	12.3276	42.5371	0	3.51106
**Residual**	92.4485	319	0.289807	--	--	0.538338
**Botanical Origin L-K**						
**Total corr.**	339	339	1	--	--	1
**Regression**	165.311	20	8.26557	15.1807	1.90984e-35	2.87499
**Residual**	173.689	319	0.544478	--	--	0.737888
**Botanical Origin MAN**						
**Total corr.**	339	339	1	--	--	1
**Regression**	170.762	20	8.53809	16.1893	1.57618e-37	2.922

**TABLE S4 | ** Permutation test validation of the OPLS-DA model with 500 permutations was performed. The intercepts of the regression lines for the permuted R^2Y and Q^2 values are reported. Negative Q2 intercepts confirm the robustness and statistical validity of all models, indicating a low risk of overfitting.

Cultivar (Y variable)	R^2Y Intercept	Q^2 Intercept
**Amfissis (AMFI)**	0.058	-0.151
**Koroneiki (KOR)**	0.059	-0.144
**Lianolia-Kerkyra (L-K)**	0.060	-0.152
**Manaki (MAN)**	0.060	-0.150
**Thasau (THA)**	0.060	-0.150

TABLE S5 | List of the NMR variables identified as discriminant by the OPLS-DA model. Only variables with VIP (Variable Importance in Projection) > 1 and |p(corr)[1]| >0.5 were considered significant contributors to class separation. The VIP, and p(corr)[1] values, the corresponding NMR chemical shift with multiplicity, the coupling constant with the assignment, and the related compound were reported.

X variable	VIP	p(corr)	δ_H (multiplicity, *J* in Hz, assignment)	Compound
2.79	1.38969	0.595096	2.79 (dd, *J* =4.1/14.3 Hz, H-18)	oleanolic acid
0.88	5.71152	0.590443	0.88 (s, H-25, H-30)	oleanolic acid/ maslinic acid
5.18	2.01406	0.57677	5.18 (t, *J* = 3.5 Hz, H-12)	oleanolic acid/ maslinic acid
0.84	4.59263	0.550202	0.84 (s, H-29)	oleanolic acid/ maslinic acid
1.68	1.70251	0.541291	1.68 (m, CH_2)	oleanolic acid
1.1	2.81839	0.513662	1.10 (s, H-27)	maslinic acid
0.68	1.56647	0.512218	0.68 (s, CH_3)	unidentified triterpenoid
3.75	4.19607	-0.501169	3.75 (m)	mannitol

TABLE S6 | Results of Tukey's HSD post-hoc tests for multiple comparisons of means for all compounds included in the violin plots. Comparisons with a p value lower than 0.05 were considered statistically significant.

Compound	Groups' Comparison	Adjusted p value
**Apigenin**	AMFI vs. KOR	0.8119
	AMFI vs. L-K	<0.0001
	AMFI vs. MAN	<0.0001
	AMFI vs. THA	<0.0001
	KOR vs. L-K	<0.0001
	KOR vs. MAN	<0.0001
	KOR vs. THA	<0.0001
	L-K vs. MAN	0.0079
	L-K vs. THA	0.6510
	MAN vs. THA	<0.0001
**Luteolin**	AMFI vs. KOR	<0.0001
	AMFI vs. L-K	<0.0001
	AMFI vs. MAN	<0.0001
	AMFI vs. THA	<0.0001
	KOR vs. L-K	0.3669
	KOR vs. MAN	0.1688

Compound	Groups' Comparison	Adjusted p value
	KOR vs. THA	<0.0001
	L-K vs. MAN	0.9998
	L-K vs. THA	0.0204
	MAN vs. THA	0.0019
**Quercetin**	AMFI vs. KOR	<0.0001
	AMFI vs. L-K	0.0027
	AMFI vs. MAN	0.0068
	AMFI vs. THA	0.0013
	KOR vs. L-K	0.9980
	KOR vs. MAN	0.2811
	KOR vs. THA	0.9987
	L-K vs. MAN	0.8809
	L-K vs. THA	>0.9999
	MAN vs. THA	0.8417
**Succinic acid**	AMFI vs. KOR	0.0002
	AMFI vs. L-K	<0.0001
	AMFI vs. MAN	0.0056
	AMFI vs. THA	0.2217
	KOR vs. L-K	0.0184
	KOR vs. MAN	>0.9999
	KOR vs. THA	0.8159
	L-K vs. MAN	0.0485
	L-K vs. THA	0.0168
	MAN vs. THA	0.9161
**Oleanolic acid**	AMFI vs. KOR	<0.0001
	AMFI vs. L-K	<0.0001
	AMFI vs. MAN	<0.0001
	AMFI vs. THA	<0.0001
	KOR vs. L-K	<0.0001
	KOR vs. MAN	0.7932
	KOR vs. THA	0.2174
	L-K vs. MAN	<0.0001
	L-K vs. THA	<0.0001
	MAN vs. THA	0.8186
**Maslinic acid**	AMFI vs. KOR	<0.0001
	AMFI vs. L-K	<0.0001
	AMFI vs. MAN	<0.0001
	AMFI vs. THA	<0.0001
	KOR vs. L-K	<0.0001
	KOR vs. MAN	0.1715
	KOR vs. THA	0.0162
	L-K vs. MAN	<0.0001

Compound	Groups' Comparison	Adjusted p value
	L-K vs. THA	0.2789
	MAN vs. THA	0.0005
**Asiatic acid**	AMFI vs. KOR	<0.0001
	AMFI vs. L-K	<0.0001
	AMFI vs. MAN	<0.0001
	AMFI vs. THA	<0.0001
	KOR vs. L-K	0.0005
	KOR vs. MAN	<0.0001
	KOR vs. THA	0.1688
	L-K vs. MAN	<0.0001
	L-K vs. THA	<0.0001
	MAN vs. THA	0.7844
**Mannitol**	AMFI vs. KOR	0.8657
	AMFI vs. L-K	<0.0001
	AMFI vs. MAN	0.9980
	AMFI vs. THA	<0.0001
	KOR vs. L-K	<0.0001
	KOR vs. MAN	0.5296
	KOR vs. THA	<0.0001
	L-K vs. MAN	<0.0001
	L-K vs. THA	0.8444
	MAN vs. THA	<0.0001
**Oleuropein**	AMFI vs. KOR	0.3371
	AMFI vs. L-K	<0.0001
	AMFI vs. MAN	0.6976
	AMFI vs. THA	<0.0001
	KOR vs. L-K	<0.0001
	KOR vs. MAN	0.9976
	KOR vs. THA	<0.0001
	L-K vs. MAN	<0.0001
	L-K vs. THA	0.9910
	MAN vs. THA	<0.0001
**Verbascoside**	AMFI vs. KOR	0.0025
	AMFI vs. L-K	0.9684
	AMFI vs. MAN	0.0044
	AMFI vs. THA	0.0338
	KOR vs. L-K	0.1614
	KOR vs. MAN	0.9445
	KOR vs. THA	0.9932
	L-K vs. MAN	0.1148
	L-K vs. THA	0.2603
	MAN vs. THA	>0.9999

Compound	Groups’ Comparison	Adjusted p value
**Ligstroside**	AMFI vs. KOR	<0.0001
	AMFI vs. L-K	<0.0001
	AMFI vs. MAN	<0.0001
	AMFI vs. THA	<0.0001
	KOR vs. L-K	0.8982
	KOR vs. MAN	0.9996
	KOR vs. THA	0.0437
	L-K vs. MAN	0.8978
	L-K vs. THA	0.0552
	MAN vs. THA	0.1646

**TABLE S7 | ** Tentatively identified compounds with UPLC-HRMS and HRMS/MS. Compounds are sorted based on their retention time (RT). Molecular formula, precursor ion, RDBeq value, fragments (above 10% relative abundance), and chemical category are also presented.

Rt (min)	Compound	Molecular formula	Experimental *m/z* [M−H]⁻	RDBeq	Δm (ppm)	HRMS/MS negative mode	Experimental *m/z* [M−H]⁺	RDBeq	Δm (ppm)	HRMS/MS positive mode	Chemical category
0.91	D-Mannitol	$C_6H_{14}O_6$	181.0717	0.5	-0.339	59.0141 (100), 71.0142 (85), 89.0249 (66), 101.0249 (63), 181.0730 (39), 73.0301 (22), 85.0300 (15)	183.0863	-0.5	-0.080	69.0335 (100), 83.0491 (34), 85.0284 (23), 57.0335 (20), 99.0440 (16), 55.0542 (15), 101.0596 (15), 61.0284 (15), 111.0440 (13), 71.0491 (12), 129.0546 (11), 87.0441 (10)	Polyol
0.94	Quinic acid	$C_7H_{12}O_6$	191.0560	2.5	-0.583	191.0560 (100), 85.0295 (27)	193.0707	1.5	0.183	95.0492 (100), 111.0442 (99, 129.0547 (71), 69.0335 (54), 83.0492 (46), 139.0389 (37), 147.0655 (16), 93.0335 (13), 67.0543 (13), 65.0387 (12), 81.0335 (12)	Polyol
1.82	Loganic acid	$C_{16}H_{24}O_{10}$	375.1292	5.5	-1.253	59.0142 (100), 69.0351(79), 213.0782 (61), 89.0252 (48), 113.0254 (47), 107.0511 (45), 71.0144 (44), 101.0253 (33), 151.0777 (24), 169.0885 (21), 119.0357 (17), 125.0619 (15)	-	-	-	-	Iridoid
2.43	Hydroxytyrosol hexoside	$C_{14}H_{20}O_8$	315.1084	5.5	-0.447	153.0569 (100), 123.0461 (51),	-	-	-	-	Phenol

Rt (min)	Compound	Molecular formula	Experimental *m/z* [M−H]⁻	RDBeq	Δm (ppm)	HRMS/MS negative mode	Experimental *m/z* [M−H]⁺	RDBeq	Δm (ppm)	HRMS/MS positive mode	Chemical category
						59.0143 (23), 71.0145 (10)					
2.59	Oleoside	$C_{16}H_{22}O_{11}$	389.1087	6.5	-0.603	59.0142 (100), 121.0666 (62), 89.0249 (39), 69.0350 (37), 71.0143 (37), 101.0250 (20), 183.0674 (17), 113.0251 (15), 165.0567 (14), 119.0357 (12)	391.1234	5.5	-0.225	211.0601 (100), 125.0233 (59), 151.0390 (53), 95.0491 (31), 123.0440 (22), 229.0708 (19), 85.0284 (13), 165.0546 (13), 167.0339 (12), 141.0545 (12), 77.0386 (10)	Secoiridoid
2.81	Hydroxytyrosol	$C_8H_{10}O_3$	153.0556	4.5	-0.767	123.0454 (100), 153.0556 (18)	-	-	-	-	Phenol
6.82	Secologanoside	$C_{16}H_{22}O_{11}$	389.1087	6.5	-0.603	69.0350 (100), 59.0142 (91), 121.0666 (38), 71.0143 (26), 89.0249 (24), 95.0503 (19), 101.0250 (16), 165.0567 (14), 113.0251 (10)	391.1234	5.5	-0.225	151.0389 (100), 95.0492 (29), 123.0439 (16), 107.0491 (13), 51.0229 (12), 211.0596 (12)	Secoiridoid
7.13	Caffeic acid di-hexoside	$C_{21}H_{30}O_{13}$	489.1609	7.5	-0.949	145.0302 (100), 163.0409 (50), 119.0508 (22), 117.0352 (17), 205.0517 (14), 265.0732 (11)	491.1758	6.5	-0.239	147.0440 (100), 65.0385 (16),	Phenol
8.10	Oleoside methyl ester	$C_{17}H_{24}O_{11}$	403.1241	6.5	-1.202	59.0143 (100), 71.0144 (41), 89.0251 (34), 101.0252 (29), 113.0253 (14), 69.0351 (12), 119.0360 (10)	405.1393	5.5	0.400	165.0547 (100), 151.0392 (97), 95.0492 (49), 57.0314 (42)	Secoiridoid
8.79	Decarboxyl elenolic acid derivative	$C_{16}H_{26}O_{10}$	377.1449	4.5	-1.114	153.0922 (100), 197.0820 (81), 59.0139 (17)	379.1599	3.5	0.070	199.0964 (100), 109.1013 (33), 135.0808 (33), 81.0699 (32),	Secoiridoid

Rt (min)	Compound	Molecular formula	Experimental *m/z* [M−H]⁻	RDBeq	Δm (ppm)	HRMS/MS negative mode	Experimental *m/z* [M−H]⁺	RDBeq	Δm (ppm)	HRMS/MS positive mode	Chemical category
										153.0913 (30), 107.0853 (18), 181.0858 (13), 79.0544 (12)	
8.85	Apigenin di-hexoside	$C_{27}H_{30}O_{15}$	593.1510	13.5	-0.326	353.0666 (100), 383.0773 (60), 473.1090 (58), 593.1516 (46), 297.0768 (28), 503.1201 (20), 325.0714 (19), 365.0668 (12), 296.0690 (11)	595.1654	12.5	-0.582	325.0707 (100), 379.0814 (67), 337.0709 (64), 295.0601 (49), 409.0916 (39), 391.0813 (36), 307.0600 (35), 349.0708 (35), 403.0817 (30), 421.0927 (27), 65.0386 (27), 439.1021 (26), 355.0814 (26), 427.1035 (26), 457.1150 (23), 361.0708 (22), 351.0864 (20), 481.1128 (17), 95.0491 (16), 321.0757 (15), 121.0283 (13), 91.0543 (12), 523.1230 (12), 335.0921 (12), 365.1022 (11), 375.0858 (11)	Flavonoid
9.36	Luteolin/kaempferol di-hexoside – isomer 1	$C_{27}H_{30}O_{16}$	609.1457	13.5	-0.670	285.0403 (100), 447.0931 (84), 284.0323 (36)	611.1603	12.5	-0.591	287.0552 (100)	Flavonoid
9.36	Hydrated derivative of decarboxylelenolic acid	$C_{10}H_{16}O_5$	215.0923	3.5	-0.70	153.0929 (100), 141.0928 (53), 215.0925 (37), 171.1035 (36), 125.0979 (23), 197.0825 (21), 219.0818 (17), 185.0825 (15)	217.1071	2.5	0.230	81.0698 (100), 199.0963 (26), 135.0804 (26), 79.0542 (24), 109.1012 (23), 67.0542 (19), 107.0855 (19),	Secoiridoid

Rt (min)	Compound	Molecular formula	Experimental *m/z* [M−H]⁻	RDBeq	Δm (ppm)	HRMS/MS negative mode	Experimental *m/z* [M−H]⁺	RDBeq	Δm (ppm)	HRMS/MS positive mode	Chemical category
										153.0912 (14), 85.0284 (12)	
9.61	Secoxyloganin	$C_{17}H_{24}O_{11}$	403.1241	6.5	-1.202	59.0138 (100), 121.0294 (99), 101.0243 (44), 71.0138 (42), 95.0502 (36), 89.0243 (34), 69.0346 (25), 139.0035 (24), 165.0553 (22), 123.0451 (19), 111.0088 (16), 113.0245 (13), 119.0351 (12), 67.0189 (12), 219.0818 (11)	405.1393	5.5	0.400	165.0547 (100), 151.0388 (90), 95.0491 (47), 123.0441 (25), 193.0489 (20), 51.0228 (14), 65.0387 (12)	Secoiridoid
9.80	Phenethyl primeveroside	$C_{19}H_{28}O_{10}$	415.1607	6.5	-0.651	59.0140 (100), 89.0248 (90), 71.0141 (48), 99.0092 (29), 101.0248 (29), 73.0298 (19), 149.0456 (13)	-	-	-	-	Phenol
10.29	Luteolin/kaempferol di-hexoside – isomer 2	$C_{27}H_{30}O_{16}$	609.1453	13.5	-1.39	285.0400 (100), 447.0926 (64), 284.0323 (21)	611.1603	12.5	-0.591	287.0549 (100), 449.1076 (28)	Flavonoid
10.50	Demethyl oleuropein	$C_{24}H_{30}O_{13}$	525.1611	10.5	-0.503	59.0138 (100), 121.0658 (89), 69.0346 (65), 165.0556 (50), 209.0455 (38), 71.0138 (30), 89.0244 (28), 101.0244 (26), 139.0036 (26), 113.0244 (20), 95.0502 (19), 139.0764 (18), 389.1078 (18), 121.0295 (12),	-		-	-	Secoiridoid

Rt (min)	Compound	Molecular formula	Experimental *m/z* [M−H]⁻	RDBeq	Δm (ppm)	HRMS/MS negative mode	Experimental *m/z* [M−H]⁺	RDBeq	Δm (ppm)	HRMS/MS positive mode	Chemical category
						183.0659 (12), 119.0348 (12), 123.0450 (10)					
10.66	Taxifolin	$C_{15}H_{12}O_7$	303.0509	10.5	-0.416	125.0244 (100), 285.0403 (30), 57.0345 (18), 175.0400 (17), 177.0193 (13), 217.0505 (12), 151.0400 (11), 199.0401 (10)	305.0655	9.5	-0.260	153.0182 (100), 149.0233 (92), 231.0651 (92), 123.0440 (73), 259.0600 (66), 167.0338 (26), 51.0229 (18), 195.0286 (16), 65.0385 (15), 241.0495 (15), 95.0491 (15), 287.0551 (15)	Flavonoid
10.70	Rutin	$C_{27}H_{30}O_{16}$	609.1454	13.5	-1.162	300.0271 (100), 301.0349 (35), 271.0244 (28), 609.1452 (22), 255.0294 (15)	611.1603	12.5	-0.591	303.0498 (100), 85.0284 (14)	Flavonoid
10.91	Hydroxyoleuropein	$C_{25}H_{32}O_{14}$	555.1716	10.5	-0.592	151.0399 (100), 123.0450 (22)	-	-	-	-	Secoiridoid
10.96	Quercetin hexoside – isomer 1	$C_{21}H_{19}O_{12}$	463.0879	12.5	-0.646	300.0273 (100), 301.0351 (37), 271.0246 (26), 255.0298 (12)	465.1025	11.5	-0.543	303.0499 (100), 85.0284 (10)	Flavonoid
10.97	Luteolin/kaempferol rutinoside – isomer 1	$C_{27}H_{30}O_{15}$	593.1508	13.5	-0.663	285.0402 (100), 593.1509 (19), 284.0326 (11)	595.1654	12.5	-0.582	287.0550 (100)	Flavonoid
11.18	Luteolin/kaempferol hexoside – isomer 1	$C_{21}H_{20}O_{11}$	447.0927	12.5	-1.280	285.0404 (100), 284.0327 (51), 447.0934 (22)	449.1075	11.5	-0.752	287.0547 (100)	Flavonoid
11.32	Luteolin/kaempferol rutinoside – isomer 2	$C_{27}H_{30}O_{15}$	593.1509	13.5	-0.494	285.0402 (100), 593.1508 (96), 284.0324 (69), 65.0032 (11)	595.1654	12.5	-0.582	287.0549 (100)	Flavonoid
11.43	Calceolarioside	$C_{23}H_{26}O_{11}$	477.1397	11.5	-1.121	161.0243 (100)	479.1545	10.5	-0.601	163.0389 (100), 89.0385 (18), 63.0230 (14)	Phenylpropanoid
11.43	Verbascoside – isomer 1	$C_{29}H_{36}O_{15}$	623.1973	12.5	-1.353	161.0242 (100), 461.1658 (16),	625.2125	11.5	-0.315	163.0390 (100)	Phenylpropanoid

Rt (min)	Compound	Molecular formula	Experimental *m/z* [M−H]⁻	RDBeq	Δm (ppm)	HRMS/MS negative mode	Experimental *m/z* [M−H]⁺	RDBeq	Δm (ppm)	HRMS/MS positive mode	Chemical category
						113.0242 (10), 135.0449 (10)					
11.94	Apigenin derivative 1	$C_{27}H_{30}O_{14}$	577.1556	13.5	-1.176	269.0450 (100)	579.1705	12.5	-0.573	271.0600 (100)	Flavonoid
11.98	Dihydrooleuropein	$C_{25}H_{36}O_{13}$	543.2079	8.5	-0.763	71.0138 (100), 59.0138 (79), 377.1458 (61), 101.0244 (54), 89.0244 (50), 151.0764 (40), 121.0658 (35), 113.0246 (30), 357.1186 (29), 119.0352 (26), 133.0660 (25), 313.1292 (21), 85.0296 (21), 165.0557 (17), 197.0820 (16), 219.0817 (15), 183.0666 (14), 73.0295 (12)	-	-	-	-	Secoiridoids
12.00	Verbascoside – isomer 2	$C_{29}H_{36}O_{15}$	623.1973	12.5	-1.353	161.0242 (100), 461.1658 (24), 623.1978 (21), 113.0243 (13), 135.0450 (12), 133.0294 (10)	-	-	-	-	Phenylpropanoid
12.02	Luteolin/Kaempferol rutinoside – isomer 3	$C_{27}H_{30}O_{15}$	593.1509	13.5	-0.494	285.0402 (100)	595.1654	12.5	-0.582	287.0550 (100)	Flavonoid
12.08	Quercetin deoxyhexoside	$C_{21}H_{20}O_{11}$	447.0927	12.5	-1.307	300.0274 (100), 301.0352 (63), 271.0246 (30), 255.0299 (16)	449.1075	11.5	-0.752	303.0498 (100), 85.0283 (31), 71.0491 (26)	Flavonoid
12.16	Verbascoside – isomer 3	$C_{29}H_{36}O_{15}$	623.1973	12.5	-1.353	161.0242 (100), 461.1658 (13), 133.0294 (12), 113.0243 (10)	-	-	-	-	Phenylpropanoid
12.24	Hydroxypinoresinol	$C_{26}H_{32}O_{12}$	535.1821	11.5	-0.933	59.0137 (100), 71.0138 (69), 89.0243 (63),	537.1962	10.5	-0.843	147.0805 (100), 91.0542 (32), 119.0855 (31),	Lignan

Rt (min)	Compound	Molecular formula	Experimental *m/z* [M−H]⁻	RDBeq	Δm (ppm)	HRMS/MS negative mode	Experimental *m/z* [M−H]⁺	RDBeq	Δm (ppm)	HRMS/MS positive mode	Chemical category
						295.0970 (56), 280.0736 (44), 101.0243 (40), 113.0242 (33), 325.1075 (30), 355.1176 (30), 151.0398 (29), 119.0348 (28), 175.0396 (27), 265.0504 (22)				165.0551 (11), 65.0386 (10)	
12.24	Luteolin/kaempferol hexoside – isomer 2	$C_{21}H_{20}O_{11}$	447.0927	12.5	-1.280	285.0400 (100)	449.1075	11.5	-0.752	287.0549 (100)	Flavonoid
12.29	Quercetin hexoside – isomer 2	$C_{21}H_{19}O_{12}$	463.0879	12.5	-0.646	301.0350 (100), 151.0035 (41), 178.9984 (20), 65.0032 (12)	465.1025	11.5	-0.543	303.0499 (100)	Flavonoid
12.31	Diosmin – isomer 1	$C_{28}H_{32}O_{15}$	607.1661	13.5	-1.224	299.0557 (100); 284.0323 (44)	609.1814	12.5	-0.651	301.0705 (100), 286.0472 (10)	Flavonoid
12.32	Apigenin derivative 2	$C_{27}H_{30}O_{14}$	577.1556	13.5	-1.176	269.0451 (100), 268.0376 (21), 577.1558 (12)	579.1705	12.5	-0.573	271.0599 (100)	Flavonoid
12.33	Apigenin hexoside	$C_{21}H_{20}O_{10}$	431.0979	12.5	-1.090	268.0375 (100), 431.0982 (46), 269.0454 (31)	433.1127	11.5	-0.515	271.0601 (100)	Flavonoid
12.46	Oleuropein hexoside	$C_{31}H_{42}O_{18}$	701.2292	11.5	-0.909	111.0087 (100), 121.0294 (97), 307.0822 (94), 95.0502 (69), 139.0036 (68), 275.0924 (64), 539.1760 (60), 275.0561 (56), 101.0244 (54), 149.0243 (51), 371.0982 (45), 223.0609 (40), 139.0399 (34), 165.0555 (34), 89.0244 (33), 127.0400 (29), 113.0244 (28),	-	-	-	-	Secoiridoid

Rt (min)	Compound	Molecular formula	Experimental *m/z* $[M-H]^-$	RDBeq	Δm (ppm)	HRMS/MS negative mode	Experimental *m/z* $[M-H]^+$	RDBeq	Δm (ppm)	HRMS/MS positive mode	Chemical category
						437.1093 (25),<br>179.0561 (23),<br>403.1244 (17),<br>119.0348 (17),<br>377.1248 (13),<br>123.0452 (12)					
12.66	Diosmin – isomer 2	$C_{28}H_{32}O_{15}$	607.1661	13.5	-1.16	299.0556 (100);<br>284.0323 (48),<br>283.0244 (19),<br>607.1661 (13),<br>255.0296 (12)	609.1814	12.5	-0.651	301.0705 (100),<br>286.0472 (16)	Flavonoid
12.73	Methyl-luteolin hexoside – isomer 1	$C_{22}H_{22}O_{11}$	461.1084	12.5	-1.159	283.0246 (100),<br>255.0298 (63),<br>461.1087 (57),<br>446.0851 (55),<br>298.0480 (30),<br>65.0033 (18),<br>63.0239 (16),<br>297.0404 (16),<br>299.0559 (15),<br>284.0322 (12)	463.1233	11.5	-0.406	301.0704 (100),<br>286.0471 (12)	Flavonoid
12.78	Nuzhenide	$C_{31}H_{42}O_{17}$	685.2346	11.5	-0.471	291.0873 (100),<br>101.0243 (79),<br>453.1398 (46),<br>259.0974 (38),<br>127.0400 (30),<br>111.0087 (26),<br>139.0400 (22),<br>89.0244 (18),<br>139.0035 (15),<br>95.0502 (11),<br>171.0298 (11),<br>121.0293 (10)	-		-	-	Secoiridoid
12.79	Acetoxypinoresinol	$C_{28}H_{34}O_{13}$	577.1921	12.5	-0.977	151.0400 (100),<br>415.1397 (69),<br>280.0739 (41),<br>295.0971 (33),<br>325.1078 (28),<br>136.0164 (22),<br>265.0506 (19),<br>181.0504 (17),	-	-	-	-	Lignan

Rt (min)	Compound	Molecular formula	Experimental *m/z* $[M-H]^-$	RDBeq	Δm (ppm)	HRMS/MS negative mode	Experimental *m/z* $[M-H]^+$	RDBeq	Δm (ppm)	HRMS/MS positive mode	Chemical category
						343.1185 (16),<br>235.0609 (13),<br>193.0503 (12)					
12.83	Methyl-luteolin hexoside – isomer 2	$C_{22}H_{22}O_{11}$	461.1084	12.5	-1.159	299.0557 (100),<br>298.0479 (80),<br>283.0244 (58),<br>297.0401 (47),<br>284.0321 (34),<br>255.0296 (26),<br>461.1082 (20),<br>63.0239 (16),<br>65.0032 (16),<br>269.0451 (14)	463.1233	11.5	-0.406	301.0706 (100),<br>286.0474 (21)	Flavonoid
12.89	Luteolin/kaempferol hexoside – isomer 3	$C_{21}H_{20}O_{11}$	447.0927	12.5	-1.280	285.0400 (100)	449.1075	11.5	-0.752	287.0549 (100)	Flavonoid
13.29	Methoxyoleuropein – isomer 1	$C_{26}H_{34}O_{14}$	569.1870	10.5	-1.017	151.0399 (100),<br>101.0243 (23),<br>89.0243 (22),<br>71.0138 (21),<br>123.0450 (19),<br>223.0611 (13),<br>95.0502 (10)	-		-	-	Secoiridoid
13.37	Methoxyoleuropein – isomer 2	$C_{26}H_{34}O_{14}$	569.1870	10.5	-1.017	151.0399 (100),<br>89.0243 (18),<br>101.0243 (17),<br>123.0450 (17),<br>71.0137 (14),<br>223.0611 (11)	-		-	-	Secoiridoid
13.48	Oleuropein – isomer 1	$C_{25}H_{32}O_{13}$	539.1764	10.5	-1.139	59.0138 (100),<br>95.0501 (85),<br>101.0243 (69),<br>89.0243 (61),<br>139.0399 (60),<br>149.0242 (60),<br>71.0138 (56),<br>111.0087 (43),<br>307.0823 (37),<br>275.0559 (37),<br>275.0922 (35),<br>68.9981 (34),<br>139.0036 (32),	-	-	-	-	Secoiridoid

Rt (min)	Compound	Molecular formula	Experimental *m/z* [M−H]⁻	RDBeq	Δm (ppm)	HRMS/MS negative mode	Experimental *m/z* [M−H]⁺	RDBeq	Δm (ppm)	HRMS/MS positive mode	Chemical category
						113.0243 (30), 69.0345 (26), 119.0349 (24), 223.0610 (23), 121.0294 (22), 127.0399 (21), 67.0189 (13), 147.0449 (11)					
13.84	Oleuropein – isomer 2	$C_{25}H_{32}O_{13}$	539.1764	10.5	-1.139	59.0138 (100), 95.0501 (77), 101.0243 (65), 149.0242 (63), 71.0138 (59), 139.0399 (52), 89.0243 (52), 111.0087 (34), 139.0036 (30), 275.0922 (29), 68.9981 (28), 113.0243 (27), 275.0555 (26), 307.0817 (25), 147.0449 (25), 165.0555 (24), 223.0610 (20), 197.0818 (20), 121.0294 (19), 119.0349 (19), 69.0345 (15), 127.0399 (13), 191.0349 (11)	-	-	-	-	Secoiridoid
13.98	Fraxamoside	$C_{25}H_{30}O_{13}$	537.1608	11.5	-1.050	151.0399 (100), 123.0450 (23)	539.1757	10.5	-0.403	165.0546 (100), 151.0389 (90), 95.0491 (49), 123.0440 (28), 193.0495 (14), 135.0441 (13)	Secoiridoid
14.12	Oleuropein – isomer 3	$C_{25}H_{32}O_{13}$	539.1764	10.5	-1.139	95.0501 (100), 59.0138 (96), 101.0243 (90), 121.0294 (76),	541.1913	9.5	-0.494	137.0597 (100), 65.0386 (16), 91.0542 (15),	Secoiridoid

Rt (min)	Compound	Molecular formula	Experimental *m/z* [M−H]⁻	RDBeq	Δm (ppm)	HRMS/MS negative mode	Experimental *m/z* [M−H]⁺	RDBeq	Δm (ppm)	HRMS/MS positive mode	Chemical category
						111.0087 (71), 89.0243 (69), 139.0037 (61), 149.0242 (56), 71.0138 (56), 139.0400 (50), 68.9981 (40), 69.0345 (36), 113.0243 (34), 119.0349 (32), 127.0399 (29), 165.0555 (27), 67.0189 (25), 123.0453(16), 95.0138 (13), 147.0449 (12)				165.0546 (11), 151.0390 (10)	
14.36	Eriodictyol	$C_{15}H_{12}O_6$	287.0558	10.5	-1.085	135.0450 (100), 151.0035 (79), 65.0032 (15), 107.0138 (14)	289.0708	9.5	0.468	163.0390 (100), 153.0182 (90), 289.0706 (65), 89.0386 (19), 63.0229 (11)	Flavonoid
14.76	Luteolin	$C_{15}H_{10}O_6$	285.0402	11.5	-0.917	285.0401 (100), 133.0294 (15)	287.0550	10.5	-0.051	287.0550 (100)	Flavonoid
14.76	Quercetin	$C_{15}H_{10}O_7$	301.0351	11.5	-0.916	151.0035 (100), 301.0351 (70), 178.9984 (40), 121.0294 (25), 65.0032 (22), 107.0137 (22)	303.0498	10.5	-0.426	303.0498 (100)	Flavonoid
14.89	Ligstroside – isomer 1	$C_{25}H_{32}O_{12}$	523.1816	10.5	-0.955	291.0871 (100), 101.0243 (74), 127.0399 (37), 68.9981 (31), 111.0086 (23), 69.0345 (15), 171.0298 (13), 139.0035 (13), 259.0975 (13), 59.0137 (12), 67.0188 (11)	525.1965	9.5	-0.291	121.0647 (100), 95.0490 (13)	Secoiridoid

Rt (min)	Compound	Molecular formula	Experimental *m/z* [M−H]$^-$	RDBeq	Δm (ppm)	HRMS/MS negative mode	Experimental *m/z* [M−H]$^+$	RDBeq	Δm (ppm)	HRMS/MS positive mode	Chemical category
15.47	Ligstroside – isomer 2	$C_{25}H_{32}O_{12}$	523.1816	10.5	-0.955	291.0871 (100), 101.0243 (82), 59.0137 (46), 68.9981 (36), 127.0400 (35), 71.0138 (26), 89.0244 (25), 111.0087 (23), 139.0400 (19), 259.0974 (19), 139.0035 (16), 67.0189 (15), 171.0299 (14), 69.0346 (11), 95.0502 (10)	525.1965	9.5	-0.291	121.0647 (100), 165.0546 (16), 95.0491 (15)	Secoiridoid
16.45	Apigenin	$C_{15}H_{10}O_5$	269.0452	11.5	-1.289	269.0453 (100); 117.0345 (21), 151.0035 (12)	271.0600	10.5	-0.369	271.0600 (100)	Flavonoid
17.00	Diosmetin	$C_{16}H_{12}O_6$	299.0559	11.5	-0.707	284.0322 (100); 299.0557 (27); 256.0375 (16)	301.0705	10.5	-0.547	301.0705 (100), 286.0471 (43)	Flavonoid
17.38	Trihydroxyoctadecadienoic acid	$C_{18}H_{32}O_5$	327.2176	3.5	-0.297	211.1338 (100), 327.2174 (72), 171.1025 (53), 229.1443 (48), 85.0294 (28), 183.1390 (23), 97.0657 (21), 221.1183 (12), 291.1954 (11)	-	-	-	-	Fatty acid
17.89	Oleuropein aglycon	$C_{19}H_{22}O_8$	377.1239	9.5	-0.771	95.0502 (100), 111.0087 (81), 149.0243 (57), 139.0400 (56), 139.0036 (49), 101.0243 (46), 68.9982 (36), 127.0400 (34), 59.0138 (25), 67.0189 (23), 121.0294 (17),	379.1386	8.5	-0.380	137.0597 (100), 91.0542 (21), 65.0386 (17), 139.0390 (11)	Secoiridoid

Rt (min)	Compound	Molecular formula	Experimental *m/z* [M−H]⁻	RDBeq	Δm (ppm)	HRMS/MS negative mode	Experimental *m/z* [M−H]⁺	RDBeq	Δm (ppm)	HRMS/MS positive mode	Chemical category
						69.0345 (16), 275.0559 (15), 147.0451 (11)					
18.07	Trihydroxyoctadecanoic acid	$C_{18}H_{36}O_5$	331.2488	1.5	-0.596	331.2487 (100), 313.2384 (11)	-	-	-	-	Fatty acid
19.03	Dihydroxypalmitic acid	$C_{16}H_{32}O_4$	287.2226	1.5	-0.636	287.2226 (100)	-	-	-	-	Fatty acid
21.99	Dihydroxyoctadecenoic acid	$C_{18}H_{34}O_4$	313.2383	2.5	-0.424	313.2383 (100), 295.2277 (13)	315.2529	1.5	-0.273	95.0857 (100), 81.0699 (99), 67.0542 (80), 109.1011 (55), 279.2319 (36), 55.0542 (33), 93.0698 (29), 69.0697 (28), 123.1168 (27), 261.2212 (25), 121.1011 (23), 79.0542 (23), 135.1168 (20), 91.0543 (18), 161.1324 (15), 97.1010 (14), 95.0491 (12)	Fatty acid
23.36	Hydroxy-octadecatrienoic acid – isomer 1	$C_{18}H_{30}O_3$	293.2122	4.5	-0.062	235.1702 (100), 275.2014 (87), 293.2119 (41)	-	-	-	-	Fatty acid
23.44	Hydroxy-octadecatrienoic acid – isomer 2	$C_{18}H_{30}O_3$	293.2122	4.5	-0.062	293.2123 (100), 195.1392 (39), 223.1339 (36), 275.2017 (24), 196.1469 (13)	-	-	-	-	Fatty acid
23.59	Oxo-octadecatrienoic acid	$C_{18}H_{28}O_3$	291.1966	5.5	0.111	291.1966 (100), 185.1182 (11)	293.2111	4.5	-0.073	219.1744 (100), 95.0854 (33), 91.0542 (29), 57.0335 (23), 93.0698 (22), 67.0542 (21), 79.0541 (20), 95.0492 (18), 55.0543 (16),	Fatty acid

Rt (min)	Compound	Molecular formula	Experimental *m/z* [M−H]⁻	RDBeq	Δm (ppm)	HRMS/MS negative mode	Experimental *m/z* [M−H]⁺	RDBeq	Δm (ppm)	HRMS/MS positive mode	Chemical category
										105.0699 (16), 81.0699 (14), 119.0855 (13), 131.0858 (13), 133.1014 (12), 275.2014 (12), 117.0701 (11), 121.1012 (10)	
23.80	Asiatic acid	$C_{30}H_{48}O_5$	487.3426	7.5	-0.611	453.3373 (100), 409.3470 (28), 451.3218 (27), 407.3316 (21), 405.3168 (12)	489.3573	6.5	-0.370	443.3520 (100), 91.0542 (24), 205.1588 (22), 187.1481 (16), 105.0698 (12), 95.0491 (11), 133.1012 (11), 119.0856 (100)	Triterpenoid
24.04	13-Hydroxyoctadecadienoic acid	$C_{18}H_{32}O_3$	295.2279	3.5	0.108	295.2277 (100), 277.2172 (48), 195.1389 (45)	-	-	-	-	Fatty acid
24.07	9(10)-Epoxyoctadecenoic acid	$C_{18}H_{32}O_3$	295.2279	3.5	0.108	277.2172 (100), 295.2277 (73), 171.1026 (72), 183.1025 (18)	-	-	-	-	Fatty acid
24.21	Glycyrrhetic acid	$C_{30}H_{46}O_4$	469.3321	8.5	-0.497	423.3266 (100), 469.3322 (75)	471.3466	7.5	-0.608	471.3466 (100), 91.0542 (43), 189.1637 (42), 425.3411 (37), 175.1481 (29), 119.0854 (28), 217.1585 (26), 105.0698 (24), 235.1692 (21), 95.0491 (21), 95.0855 (21), 135.1168 (19), 121.1011 (19), 107.0855 (18), 79.0541 (17), 187.1480 (16), 133.1011 (16), 115.0542 (15),	Triterpenoid

Rt (min)	Compound	Molecular formula	Experimental *m/z* [M−H]$^-$	RDBeq	Δm (ppm)	HRMS/MS negative mode	Experimental *m/z* [M−H]$^+$	RDBeq	Δm (ppm)	HRMS/MS positive mode	Chemical category
										65.0385 (14), 93.0699 (14), 271.2058 (13), 81.0698 (13), 109.1010 (12), 407.3300 (12), 177.1638 (11), 55.0542 (11), 147.1169 (11)	
24.31	Maslinic acid	$C_{30}H_{48}O_4$	471.3476	7.5	-0.813	471.3479 (100)	473.3622	6.5	-0.711	203.1794 (100), 409.3466 (65), 189.1637 (44), 205.1586 (39), 177.1637 (38), 95.0854 (31), 121.1011 (28), 91.0541 (27), 187.1479 (24), 149.1322 (21), 105.0697 (20), 175.1482 (20), 107.0856 (19), 109.1012 (19), 133.1012 (18), 95.0491 (18), 119.0855 (18), 81.0700 (16), 79.0543 (15), 207.1743 (15), 135.1171 (14), 163.1484 (13), 147.1169 (13), 93.0697 (13), 191.1795 (11), 65.0386 (11), 161.1324 (11), 67.0543 (11), 55.0542 (10), 105.0442 (10)	Triterpenoid
24.33	Hydroxy-octadecenoic acid – isomer 1	$C_{18}H_{34}O_3$	297.2435	2.5	-0.061	297.2434 (100)	-	-	-	-	Fatty acid

Rt (min)	Compound	Molecular formula	Experimental *m/z* [M−H]⁻	RDBeq	Δm (ppm)	HRMS/MS negative mode	Experimental *m/z* [M−H]⁺	RDBeq	Δm (ppm)	HRMS/MS positive mode	Chemical category
24.52	Corosolic acid	$C_{30}H_{48}O_4$	471.3476	7.5	-0.813	471.3477 (100)	473.3622	6.5	-0.711	205.1587 (100), 91.0542 (55), 427.3563 (51), 189.1640 (51), 207.1744 (43), 203.1793 (34), 95.0856 (33), 95.0493 (33), 81.0698 (32), 79.0543 (28), 409.3462 (28), 121.1013 (27), 93.0700 (25), 105.0701 (23), 177.1640 (22), 147.1173 (22), 109.1011 (21), 55.0543 (21), 107.0856 (21), 67.0543 (20), 135.1171 (20), 65.0386 (19), 133.1012 (19), 147.0440 (18), 201.1640 (18), 149.1322 (18), 187.1484 (18), 161.1330 (17), 69.0699 (16), 163.1487 (16), 119.0855 (15), 219.1738 (12), 115.0542 (12), 175.1480 (11)	Triterpenoid
24.71	Hydroxy-octadecenoic acid – isomer 2	$C_{18}H_{34}O_3$	297.2435	2.5	-0.061	297.2434 (100), 279.2330 (22), 171.1027 (16)	-	-	-	-	Fatty acid
24.76	Hydroxy-octadecenoic acid – isomer 3	$C_{18}H_{34}O_3$	297.2435	2.5	-0.061	297.2434 (100), 279.2328 (25), 155.1077 (24)	-	-	-	-	Fatty acid

Rt (min)	Compound	Molecular formula	Experimental *m/z* [M−H]$^-$	RDBeq	Δm (ppm)	HRMS/MS negative mode	Experimental *m/z* [M−H]$^+$	RDBeq	Δm (ppm)	HRMS/MS positive mode	Chemical category
24.89	Hydroxyoctadecadienoic acid – isomer 1	$C_{18}H_{32}O_3$	295.2279	3.5	0.108	295.2278 (100)	-	-	-	-	Fatty acid
25.01	Hydroxyoctadecadienoic acid – isomer 2	$C_{18}H_{32}O_3$	295.2279	3.5	0.108	295.2278 (100), 155.1442 (12)	-	-	-	-	Fatty acid
25.30	3-*β*-O-*cis*-Coumaroylmaslinic acid	$C_{39}H_{54}O_6$	617.3845	13.5	-0.425	617.3847 (100), 145.0295 (71), 117.0346 (27)	619.3990	12.5	-0.510	147.0440 (100), 91.0541 (50), 189.1637 (46), 203.1793 (43), 119.0856 (29), 201.1638 (28), 95.0856 (23), 105.0699 (21), 121.1010 (21), 215.1797 (20), 147.1170 (20), 177.1641 (19), 165.0546 (19), 175.1480 (19), 133.1013 (18), 107.0857 (18), 135.1169 (15), 149.1326 (14), 161.1329 (14), 123.1167 (13), 163.1479 (13), 67.0542 (13), 437.3404 (13), 391.3366 (12), 409.3468 (12), 81.0699 (12), 93.0700 (11), 109.1010 (10)	Triterpenoid
25.43	3-*β*-O-*trans*-Coumaroylmaslinic acid	$C_{39}H_{54}O_6$	617.3845	13.5	-0.425	617.3847 (100), 145.0295 (61), 117.0346 (33)	619.3990	12.5	-0.510	147.0440 (100), 91.0541 (37), 203.1793 (32), 165.0546 (26), 189.1637 (24), 119.0856 (20), 95.0856 (18), 201.1638 (18), 215.1793 (16),	Triterpenoid

Rt (min)	Compound	Molecular formula	Experimental *m/z* [M−H]$^-$	RDBeq	Δm (ppm)	HRMS/MS negative mode	Experimental *m/z* [M−H]$^+$	RDBeq	Δm (ppm)	HRMS/MS positive mode	Chemical category
										177.1641 (15), 147.1170 (14), 105.0699 (14), 121.1010 (13), 107.0857 (13), 437.3410 (13), 175.1485 (13), 95.0491 (13), 133.1013 (12), 109.1010 (12), 135.1169 (12), 81.0699 (11)	
26.21	Betulinic acid	$C_{30}H_{48}O_3$	455.3528	7.5	-0.590	455.3531 (100)	457.3677	6.5	0.171	203.1794 (100), 189.1638 (84), 191.1794 (62), 95.0855 (56), 91.0542 (52), 135.1167 (41), 163.1483 (36), 121.1010 (31), 109.1011 (30), 411.3621 (29), 119.0854 (28), 95.0492 (25), 133.1013 (25), 81.0699 (24), 107.0855 (23), 79.0543 (23), 67.0543 (21), 105.0699 (21), 175.1483 (19), 65.0386 (18), 123.1169 (16), 93.0699 (16), 161.1325 (16), 439.3575 (15), 235.1696 (15), 149.1326 (15), 187.1480 (15), 55.0542 (14), 231.2112 (14),	Triterpenoid

Rt (min)	Compound	Molecular formula	Experimental *m/z* [M−H]$^{-}$	RDBeq	Δm (ppm)	HRMS/MS negative mode	Experimental *m/z* [M−H]$^{+}$	RDBeq	Δm (ppm)	HRMS/MS positive mode	Chemical category
										147.1172 (14), 207.1744 (13), 177.1643 (13), 457.3665 (12), 105.0447 (12), 249.1849 (11), 77.0387 (10)	
26.44	Oleanolic acid	$C_{30}H_{48}O_3$	455.3528	7.5	-0.590	455.3531 (100)	457.3677	6.5	0.171	411.3624 (100), 203.1794 (83), 163.1482 (66), 189.1638 (60), 91.0542 (59), 95.0855 (58), 121.1011 (43), 191.1794 (42), 109.1011 (40), 177.1639 (34), 81.0700 (30), 107.0856 (29), 149.1326 (28), 135.1169 (27), 119.0854 (25), 55.0542 (25), 133.1013 (25), 95.0491 (24), 105.0699 (23), 175.1480 (21), 67.0543 (20), 79.0542 (20), 147.1170 (20), 161.1325 (19), 231.2110 (17), 123.1168 (17), 393.3520 (16), 93.0700 (15), 221.1900 (14), 217.1956 (14), 187.1486 (13), 205.1950 (13), 65.0387 (11),	Triterpenoid

Rt (min)	Compound	Molecular formula	Experimental *m/z* [M−H]⁻	RDBeq	Δm (ppm)	HRMS/MS negative mode	Experimental *m/z* [M−H]⁺	RDBeq	Δm (ppm)	HRMS/MS positive mode	Chemical category
										439.3560 (11), 105.0448 (10)	
26.91	Linoleic acid	$C_{18}H_{32}O_2$	279.2330	3.5	0.166	279.2330 (100)	-	-	-	-	Fatty acid
27.73	Oleic acid	$C_{18}H_{34}O_2$	281.2486	4.5	-0.013	281.2486 (100)	-	-	-	-	Fatty acid
